# Glutarate regulates T cell metabolism and anti-tumour immunity

**DOI:** 10.1038/s42255-023-00855-2

**Published:** 2023-08-21

**Authors:** Eleanor Minogue, Pedro P. Cunha, Brennan J. Wadsworth, Guinevere L. Grice, Shiv K. Sah-Teli, Rob Hughes, David Bargiela, Alessandro Quaranta, Javier Zurita, Robin Antrobus, Pedro Velica, Laura Barbieri, Craig E. Wheelock, Peppi Koivunen, James A. Nathan, Iosifina P. Foskolou, Randall S. Johnson

**Affiliations:** 1 Department of Physiology, Development and Neuroscience, University of Cambridge, Cambridge, UK; 2 Department of Cell and Molecular Biology, Karolinska Institutet, Stockholm, Sweden; 3 Cambridge Institute of Therapeutic Immunology & Infectious Disease, Department of Medicine, Jeffrey Cheah Biomedical Centre, Cambridge Biomedical Campus, University of Cambridge, Cambridge, UK; 4 Biocenter Oulu, Faculty of Biochemistry and Molecular Medicine, Oulu Centre for Cell-Matrix Research, University of Oulu, Oulu, Finland; 5 Unit of Integrative Metabolomics, Institute of Environmental Medicine, Karolinska Institutet, Stockholm, Sweden; 6 Cambridge Institute for Medical Research, University of Cambridge, Cambridge, UK; 7 Department of Respiratory Medicine and Allergy, Karolinska University Hospital, Stockholm, Sweden

**Keywords:** Immunization, Metabolomics, Metabolism, Tumour immunology

## Abstract

T cell function and fate can be influenced by several metabolites: in some cases, acting through enzymatic inhibition of α-ketoglutarate-dependent dioxygenases, in others, through post-translational modification of lysines in important targets. We show here that glutarate, a product of amino acid catabolism, has the capacity to do both, and has potent effects on T cell function and differentiation. We found that glutarate exerts those effects both through α-ketoglutarate-dependent dioxygenase inhibition, and through direct regulation of T cell metabolism via glutarylation of the pyruvate dehydrogenase E2 subunit. Administration of diethyl glutarate, a cell-permeable form of glutarate, alters CD8^+^ T cell differentiation and increases cytotoxicity against target cells. In vivo administration of the compound is correlated with increased levels of both peripheral and intratumoural cytotoxic CD8^+^ T cells. These results demonstrate that glutarate is an important regulator of T cell metabolism and differentiation with a potential role in the improvement of T cell immunotherapy.

## Abstract (web summary)

Minogue et al. show that glutarate, a metabolite derived from tryptophan catabolism, has the ability to shape anti-tumour T cell responses by modulating pyruvate handling and alpha-ketoglutarate-dependent dioxygenases.

## Main

T cell activation initiates the process whereby quiescent, naive T cells differentiate into rapidly proliferating effector cells. This transition involves a profound and dynamic metabolic reprograming. After the resolution of an immune response, some T cells transition to memory cells: these cells are primed for rapid recall responses. Most of the remaining T cells become exhausted and then die through apoptosis^1,2^. T cell differentiation, memory cell formation and T cell survival are regulated by many intrinsic factors, including transcriptional and epigenetic regulation^1,3–5^. These processes are also regulated by extrinsic factors, including both nutrient and oxygen availability^6–10^.

In recent years, our laboratory and others illustrated the importance of the S enantiomer of 2-hydroxyglutarate (S-2HG) in determining T cell fate^11–13^. We showed that supplementation of T cell cultures with esterified forms of S-2HG enhances T cell immunotherapy in preclinical models, where it acts by promoting the development of central memory T (T_CM_) cells. 2HG and the metabolites succinate and fumarate are structural analogues of α-ketoglutarate (αKG) and all can act as competitive inhibitors of αKG-dependent dioxygenases (αKGDDs)^14–18^. These enzymes have essential roles in many cellular processes, and there are currently over 60 identified^19–21^. S-2HG itself has been shown to exert many of its modulation of T cell function via competitive inhibition of specific αKGDDs^11^.

In this study, we sought to determine whether molecules structurally similar to S-2HG might exert similar effects on T cell differentiation and function. We describe our finding that glutarate is found at significant levels in activated T cells and is a potent inhibitor of several αKGDDs. In addition, we found that glutarate can strongly influence cellular metabolism and T cell differentiation.

Glutarate, in its coenzyme A (CoA) form, is an important intermediate of both tryptophan and lysine catabolism^22,23^. Glutarate and glutaryl-CoA arise during the final stages of the kynurenine pathway, a catabolic process which has many demonstrated roles in immunoregulation; however, curiously, glutarate has not been extensively studied in an immune context previously. Glutaryl-CoA can be broken down to either crotonyl-CoA by the enzyme glutaryl-CoA dehydrogenase (GCDH), or form glutarate^24^. The glutarate to glutaryl-CoA reaction is mediated by succinyl-CoA:glutarate-CoA transferase (SUGCT)^25^.

The ratio of crotonyl-CoA to glutarate is highly regulated, and excessive glutarate accumulation can be cytotoxic^26^. Previous work on the cellular and physiological roles of glutarate has almost exclusively focused on patients with loss of function mutations in the GCDH enzyme, which results in excessive glutarate accumulation. These patients present with the metabolic disorder glutaric aciduria type 1 (GA1). This autosomal recessive genetic disorder has varying outcomes, and these range from mild to severe developmental disorders and to seizure syndromes^26–29^.

Other than work on GA1, there is a very limited literature describing the physiological or metabolic role of glutarate. We show in this study that glutarate is present at significant levels in activated CD8^+^ T cells, and that these levels fluctuate greatly during activation. We additionally illustrate that glutarate controls the activity of the pyruvate dehydrogenase complex (PDHc) via post-translational modification glutarylation. We show that glutarylation disrupts lipoylation of the PDHc E2 subunit and reduces PDHc catalytic activity. Together, our findings highlight glutarate as an important metabolite with significant T cell modulatory capacity.

## Results

### Glutarate is an endogenous regulator of CD8^+^ T cell function

We first sought to investigate if compounds structurally related to 2HG could increase the T_CM_ population of CD8^+^ T cells. To carry out this analysis, we identified 19 structural analogues of 2HG; eight were commercially available and 11 were synthesized de novo for the purposes of this study (Table 1). We used esterified versions of most of the compounds used, so as to increase both potency and intracellular translocation. The octyl ester form of S-2HG was used as a positive control in these assays. We screened the compounds for effects on T cell differentiation at 400-µM levels (using the efficacy of S-2HG in CD8^+^ T cells as a guide^11^) in culture media during the process of activation of isolated primary murine CD8^+^ T cells.

**Table 1:** List of test compounds (plus S-2HG) used in the screen described in Fig. 1a

Commercially available			Newly synthesized	
ID	Molecular formula	Description	ID	Molecular formula
S2HG	C_13_H_25_NaO_5_	(2S)-2-hydroxyglutaric acid octyl ester sodium salt	163	C_12_H_21_NO_6_
DSB	C_8_H_14_O_4_S	1,4-diethyl 2-sulfanylbutanedioate	158	C_9_H_17_NO_4_
DTA	C_8_H_14_O_4_S	Diethyl 2,2′-thiodiacetate	166	C_12_H_20_O_6_S
DIM	C_8_H_15_NO_4_	Diethyl iminodiacetate	159	C_9_H_16_O_5_
DMA	C_8_H_14_O_5_	D-(+)-malic acid diethyl ester	164	C_12_H_19_NO_7_
DASH	C_8_H_15_NO_4_	(S)-diethyl 2-aminosuccinate hydrochloride	161	C_8_H_14_O_5_
DGA	C_9_H_17_NO_4_	L-glutamic acid diethyl ester hydrochloride	168	C_13_H_22_O_6_
EOA	C_8_H_13_NO_5_	N-(2-ethoxy-2-oxoacetyl)glycine ethyl ester	162	C_10_H_18_O_4_
DEG	C_9_H_16_O_4_	Diethyl glutarate	167	C_9_H_16_O_4_S
			165	C_12_H_20_O_7_

**Fig. 1:**
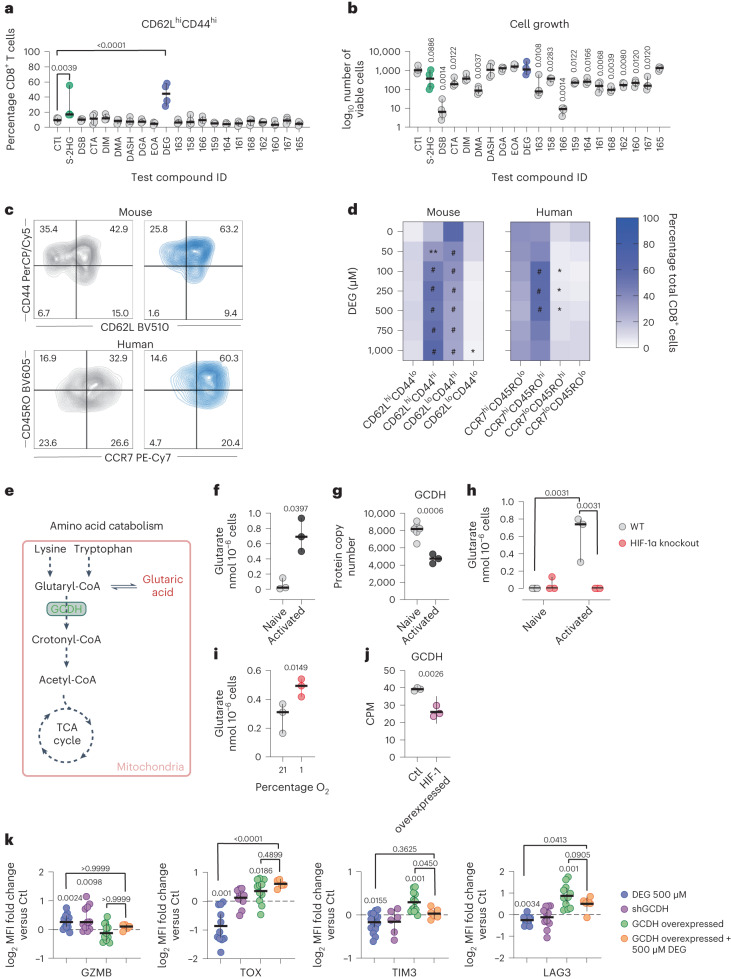
Glutarate is an endogenous regulator of CD8^+^ T cell function. **a**,**b**, Percentage of CD62L^hi^CD44^hi^ (**a**) and number of total murine CD8^+^ T cells (**b**) after 7 days of treatment with 400 µM of test compound. Ordinary one-way analysis of variance (ANOVA) relative to untreated control cells (Ctl); *n* = 4. **c**, Representative flow cytometry counter of mouse and human CD8^+^ T cells treated with or without 500 µM DEG for 7 days. Grey (left), Ctl; blue (right), DEG 500 µM. **d**, Heatmap representing the proportion of mouse CD8^+^ T cells expressing CD62L/CD44 (left) and the proportion of human CD8^+^ T cells expressing CCR7/CD45RO (right) after 7 days of treatment with increasing concentrations of DEG. Two-way ANOVA relative to Ctl; *n* = 4–5. **e**, Pathway of lysine and tryptophan catabolism (adapted from BioRender.com). **f**, Glutarate levels in naive and 72-h activated mouse CD8^+^ T cells. Two-tailed paired *t*-test; *n* = 3. **g**, GCDH protein copy number in naive or activated CD8^+^ T cells from P14 transgenic mice. Two-tailed unpaired *t*-test; *n* = 3. Data from the ImmPRes database^30^. **h**, Glutarate levels in naive or 72-h activated CD8^+^ T cells isolated from Hif-1α^*loxP*/*loxP*^ or Hif-1α^*loxP*/*loxP*^ dlck^Cre^ mice (HIF knockout). Two-way ANOVA; *n* = 3. **i**, Glutarate levels in mouse CD8^+^ T cells cultured at 21% or 1% oxygen for 24 h from day 5 after activation. Two-tailed paired *t*-test; *n* = 3. **j**, GCDH gene counts per million (CPM) in HIF-1-overexpressing mouse CD8^+^ T cells. Two-tailed unpaired *t*-test; *n* = 3^32^. **k**, Expression of markers of T cell differentiation and exhaustion in human CD8^+^ T cells after 7–10 days of treatment with or without DEG 500 µM or transduction with either shGCDH or GCDH-overexpressing vector. Expression was determined by flow cytometry and is shown as the mean fluorescence intensity (MFI) fold change relative to the untreated control (black dashed line). Two-tailed, one-sample Wilcoxon rank-sum test and ordinary one-way ANOVA. (GZMB DEG 500 µM, *n* = 13; GZMB shGCDH, *n* = 11; GZMB GCDH overexpressed, *n* = 11; GZMB GCDH overexpressed + DEG 500 µM, *n* = 5; TOX DEG 500 µM, *n* = 11; TOX shGCDH, *n* = 11; TOX GCDH overexpressed, *n* = 11; TOX GCDH overexpressed + DEG 500 µM, *n* = 5; TIM3 DEG 500 µM, *n* = 16; TIM3 shGCDH, *n* = 6; TIM3 GCDH overexpressed, *n* = 12; TIM3 GCDH overexpressed + DEG 500 µM, *n* = 6; LAG3 DEG 500 µM, *n* = 12; LAG3 shGCDH, *n* = 11; LAG3 GCDH overexpressed, *n* = 11; LAG3 GCDH overexpressed + DEG 500 µM, *n* = 6). All scatter plots show the median + the 95% confidence interval (CI), where each dot represents one donor (murine or human as indicated). *P < 0.05, **P < 0.01, ***P < 0.001, ****P < 0.0001.

**Extended Data Fig. 1:**
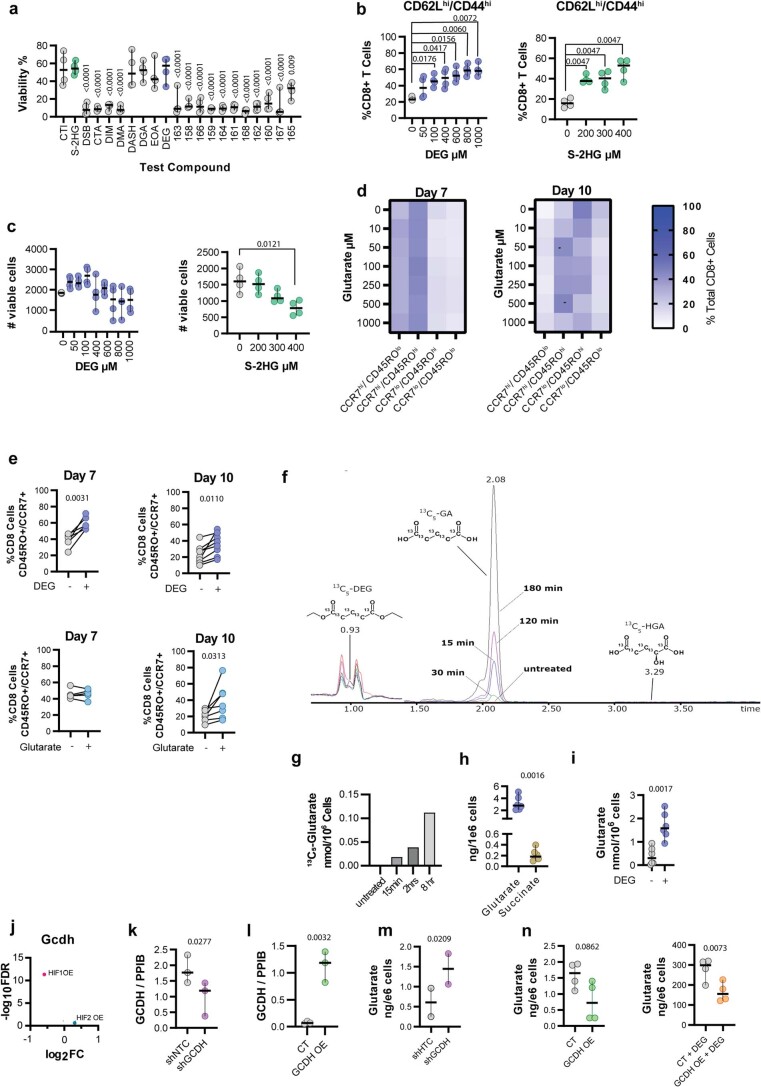
Glutarate is an endogenous regulator of CD8^+^ T cell function. **a**, Percentage viability of murine CD8^+^ T cells following 7 days of treatment with 400 µM of test compound, as determined by flow cytometry. Ordinary one-way ANOVA relative to CT; n = 4. **b**, Percentage of murine CD62L^hi^/CD44^hi^ CD8^+^ T cells following 7 days of treatment with increasing concentrations of DEG (left) or S-2HG (right). Ordinary one-way ANOVA; n = 4. **c**, Number of viable murine CD8^+^ cells following 7 days of treatment with increasing concentration of DEG (right) or S-2HG (left), as determined by counting beads and flow cytometry. Ordinary one-way ANOVA relative to CT; n = 4. **d**, Heatmap representing the proportion of human CD8^+^ T cells expressing CCR7/CD45RO following 7 or 10 days treatment with increasing concentrations of DEG. Two-way ANOVA relative to CT; n = 4-5. **e**, Percentage of CD62L^hi^/CD44^hi^ human CD8^+^ T cells human 7- or 10-days culture with DEG 500 µM or Glutarate 500 µM. Two-tailed paired t-test; DEG 7 days n = 6; DEG 10 days n = 8, Glutarate 7 days n = 5; Glutarate 10 days n = 8. **f**, Chromatograms of isotope tracing. Human CD8^+^ T cells were treated with ^13^C_5_-DEG for varying time length and mass spectrometry for glutarate was performed. **g**, Quantified ^13^C_5_-Glutarate from cells treated as described and shown as chromatograms in Extended Data Fig. 1f. **h**, Glutarate and succinate levels in activated human CD8^+^ T cells. Two-tailed paired t-test; n = 6. **i**, Glutarate levels in CD8^+^ T cells cultured -/+ DEG 500 µM for 7 days. Two-tailed paired t-test; n = 6. **j**, *Gcdh* levels in transcripts HIF1 OE (HIF1AAA) and HIF2 OE (HIF2AAA) – transduced mouse CD8^+^ T cells, relative to vector control^34^. **k**, GCDH levels in T cells transduced with an shNTC or shGCDH vectors as determined by western blot analysis. Two-tailed paired t-test; n = 3. **l**, GCDH levels in T cells transduced with a CT or GCDH overexpressing vector as determined by western blot analysis. Two-tailed paired t-test; n = 3. **m**, Glutarate levels in HEK293 cells transduced with shGCDH overexpressing vectors used in Fig. 1k and Extended Data Fig. 1k. Two-tailed paired t-test; n = 2. **n**, Glutarate levels in HEK293 cells transduced with GCDH overexpressing vectors and treated with 500 µM and used in Fig. 1k and Extended Data Fig. 1l. Two-tailed paired t-test; n = 4. All scatter plots and bar charts show median and 95% CI where each dot represents one human donor. *p < 0.05. Source data

After 7 days of culture, we assessed the abundance of CD62L^hi^CD44^hi^ (T_CM_-like) T cells by flow cytometry (Fig. 1a). We additionally determined the effect of each compound on cell growth and viability (Fig. 1b and Extended Data Fig. 1a). Of the 19 test compounds assayed, only diethyl glutarate (DEG) significantly increased the abundance of CD62L^hi^CD44^hi^ CD8^+^ T cells in our assays (Fig. 1a). Further, treatment with DEG had no negative effects on either cell viability or proliferation (Fig. 1b and Extended Data Fig. 1a). DEG increased the CD62L^hi^CD44^hi^ T_CM_ population in a dose-dependent manner, at concentrations similar to those used with treatment with octyl ester S-2HG (Extended Data Fig. 1b).

As noted, DEG did not display any negative effects on cell growth, even at 1-mM concentrations. This contrasts with octyl ester S-2HG, which decreases cell growth from 400 µM (Extended Data Fig. 1c). Further analysis of memory populations in both mouse and human CD8^+^ T cells treated with DEG revealed that after 7 days of culture, DEG increased the T_CM_ population in both mouse and human differentiating T cells (as represented by CD62L^hi^CD44^hi^ in mouse and CCR7^hi^CD45RO^hi^ in human cells), and decreased effector memory T (T_EM_) cell populations (as represented by CD62L^lo^CD44^hi^ in mouse and CCR7^lo^CD45RO^hi^ in human CD8^+^ T cells) (Fig. 1c,d).

DEG is a di-esterified form of the metabolite glutaric acid (glutarate). In the initial screen of metabolites (Figs. 1a), five compounds with diethyl-ester groups were used to increase cellular translocation, with only one, DEG, having an effect on CD8^+^ T cell differentiation. This indicates that these effects on differentiation are not likely due to treatment with diethyl group-containing compounds or the esterified ethyl groups. Additionally, we found that treatment with glutarate alone was also capable of increasing T_CM_ populations during the activation and differentiation of CD8^+^ T cells, although this was seen at higher doses after 10 days of culture (Extended Data Fig. 1d,e). This last result indicates that although exogenous glutarate may be taken up by T cells, the esterified version of glutarate is more effective at modulating CD8^+^ T cell differentiation.

To affirm that exogenous administration of DEG leads to increased intracellular glutarate levels, we performed isotope tracing of human CD8^+^ T cells treated with ^13^C_5_-labelled DEG. Tracing showed that DEG is very rapidly converted into intracellular glutarate after uptake (Extended Data Fig. 1f,g). These data indicate that processing of DEG into glutarate occurs within minutes after DEG uptake. Consistent with this finding, intracellular DEG could not be detected intracellularly after just 15 min of administration of the labelled compound (Extended Data Fig. 1f).

Glutarate is a product of tryptophan and lysine degradation, close to the terminus of the kynurenine pathway (Fig. 1e). Although a great deal is known about immunoregulatory aspects of other parts of the kynurenine pathway, there is only a very limited literature concerned with glutarate in immune cells. We thus sought to determine the amount of glutarate present in naive and activated CD8^+^ T cells. Mass spectrometry was used to determine intracellular levels of endogenous glutarate in CD8^+^ T cells: we found that glutarate levels increased substantially after T cell activation (Fig. 1f). These activation-induced increases in intracellular glutarate correlate with previously published decreases in GCDH protein copy numbers after T cell activation (Fig. 1g)^30^.

Glutarate levels in activated T cells are significantly higher than, for example, the intracellular levels of succinate, a known immunometabolite (Extended Data Fig. 1h). Using previously published CD8^+^ T cell volumes, we determined that the approximate concentration of glutarate is 0.6 mM in activated CD8^+^ T cells^11^. Treating CD8^+^ T cells with 500 µM DEG for 7 days increased measured intracellular glutarate concentrations by approximately twofold (Extended Data Fig. 1i).

T cell metabolism is greatly altered by T cell activation; a major driver of this metabolic change is the hypoxia-inducible factor 1-alpha (HIF-1α) transcription factor. HIF-1α is stabilized after CD8^+^ T cell activation even when T cells are cultured at high oxygen levels^31^, and the accumulation of a number of immunometabolites are affected by HIF-1α levels. To determine if the observed increase of glutarate levels after T cell activation was HIF-1-dependent, we assayed glutarate levels in HIF-1α proficient and HIF-1α null (HIF-1 knockout) mouse CD8^+^ T cells and found that the activation-induced increases in glutarate seen in wild-type (WT) cells is almost completely absent in HIF-1 knockout cells (Fig. 1h). This finding indicates that the HIF-1α transcription factor is essential for modulating T cell glutarate levels. We also found that culturing T cells at 1% oxygen for 24 h increased glutarate levels approximately twofold compared to culturing T cells at 21% oxygen levels (Fig. 1i). Consistent with this finding, we found that *GCDH* gene expression levels are decreased in T cells transfected with HIF-1α-overexpressing, but not HIF-2α-overexpressing T retroviral vectors (Fig. 1j and Extended Data Fig. 1j)^32^.

Given that expression of the *GCDH* encoding gene and GCDH protein levels fluctuate in CD8^+^ T cells, and that these variations correlate with changes in intracellular glutarate levels, we wished to determine the extent to which genetically modifying GCDH might influence CD8^+^ T cell phenotype by altering endogenous glutarate levels in CD8^+^ T cells. For these experiments, we undertook both silencing of GCDH (shGCDH) and overexpression of GCDH (GCDH overexpressed) with, respectively, transfection of either short hairpin RNA (shRNA) or overexpression vectors (Extended Data Fig. 1k,l). The shGCDH vector caused reduced GCDH protein levels and the GCDH overexpressed vector caused increased GCDH protein levels in T cells after transduction (Extended Data Fig. 1k,l). The shGCDH vector increased endogenous glutarate levels in HEK cells transduced with it; there were decreased levels of glutarate found in HEK cells overexpressing GCDH (Extended Data Fig. 1m). The overexpression of GCDH even reduced the elevated levels of glutarate in DEG-treated HEK cells (Extended Data Fig. 1n). Thus, these vectors enabled us to query the role of endogenous intracellular glutarate.

We found that both CD8^+^ T cells treated with DEG and CD8^+^ T cells treated with shGCDH, showed similar increases in expression of the cytotoxic effector molecule granzyme B (GZMB) (Fig. 1k). Following on from our observations that DEG alters T cell differentiation, we examined multiple exhaustion-associated markers in treated cells and found that TOX, a transcription factor known to orchestrate exhaustion^33,34^, and the exhaustion markers TIM3 and LAG3, were all downregulated in human CD8^+^ T cell cultures after 10 days of DEG treatment (Fig. 1k). In contrast, reducing glutarate levels via overexpression of GCDH (with the GCDH overexpressed vector) resulted in increased expression of TOX, TIM3 and LAG3. Supplementing GCDH-overexpressed CD8^+^ T cells with DEG counteracted the effects of GCDH overexpression on TIM3 and LAG3 (Fig. 1k). Taken together, these data illustrate that glutarate can alter CD8^+^ T cell differentiation.

### Glutarate is an inhibitor of αKGDDs

**Extended Data Fig. 2:**
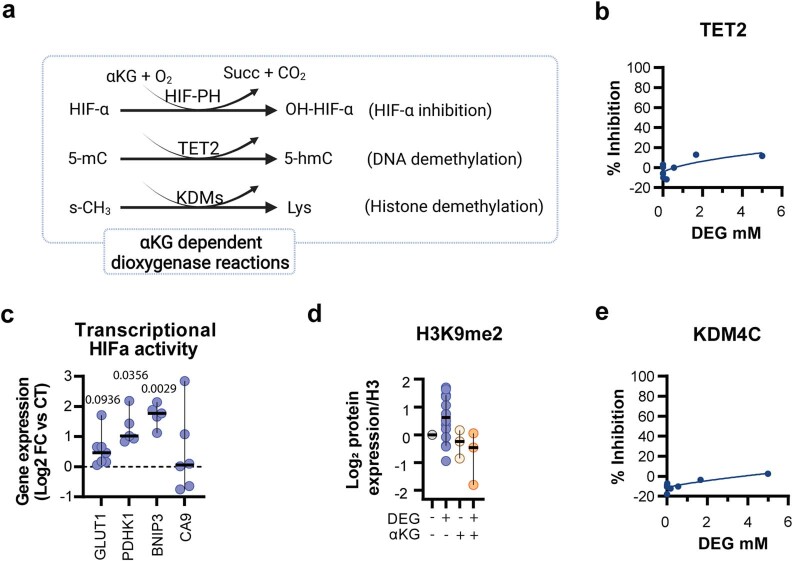
Glutarate is an inhibitor of αKGDDs. **a**, Illustration of alpha-ketoglutarate (αKG) dependent dioxygenase (αKGDD) reactions investigated in this study. *(Adapted from BioRender.com)* **b**, Cell free enzymatic inhibition assay for TET2 using increasing concentrations of DEG. **c**, qPCR analysis of CD8^+^ T cells treated with 500 µM DEG for 7 days. Each dot represents one human donor relative to untreated donor control (dotted black line), normalised to HPRT. Two-tailed one sample t-test; GLUT1 n = 8; PDHK1 n = 5; BNIP3 n = 5; CA9 n = 6. **d**, Log_2_ FC protein expression of H3K9me2 in human CD8^+^ T cells treated with +/- 500 µM DEG and 500 µM αKG for 7 days. Ordinary one-way ANOVA; DEG n = 11, αKG n = 3; αKG + DEG n = 3. **e**, Cell free enzymatic inhibition assay for TET2 using increasing concentrations of glutarate and 5x αKG than in Fig. 2I. Cell free enzymatic assay graphs show Michaelis-Menten line of best fit of at least 3 independent experiments. All scatter plots show median + 95% CI, where each dot represents one donor as indicated. Source data

Glutarate has structural similarities to 2HG, fumarate, succinate and αKG, and thus is a candidate competitive inhibitor of αKGDDs^14–18^. To determine whether this was in fact the case, we focused on three subfamilies of the most intensively studied αKGDDs: the DNA-demethylating Ten-eleven translocation methylcytosine dioxygenases (TETs), the HIF prolyl 4-hydroxylases (HIF-P4Hs) and the histone lysine demethylases (KDMs). All of these enzymatic reactions are depicted in Extended Data Fig. 2a.

**Fig. 2:**
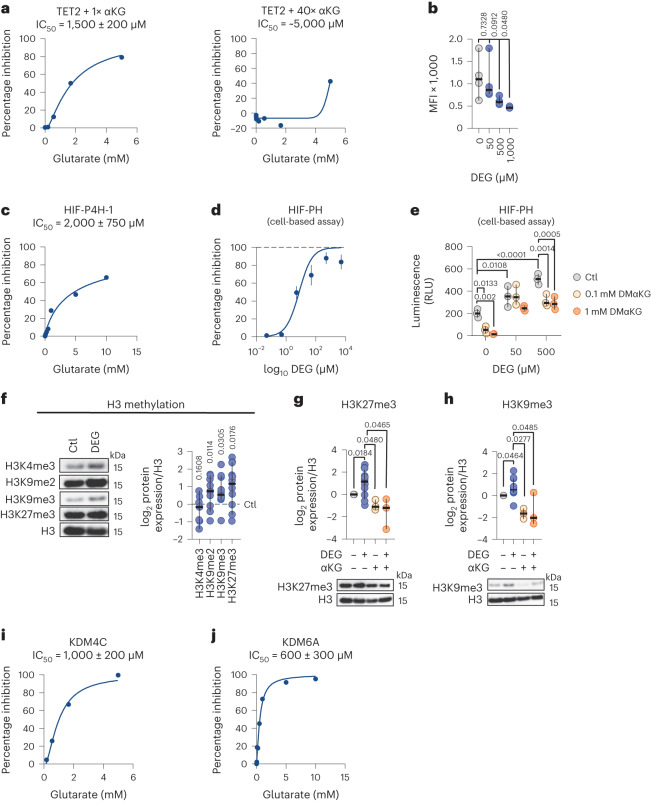
Glutarate is an inhibitor of αKG-dependent reactions. **a**, Cell-free enzymatic inhibition assay for TET2 using increasing concentrations of glutarate, using 1× IC_50_ for αKG (left) or 40× IC_50_ for αKG (right). **b**, 5hmC MFI, as determined using flow cytometry, in human CD8^+^ T cells after 7 days of treatment with increasing concentrations of DEG. Ordinary one-way ANOVA; *n* = 4. **c**, Cell-free enzymatic inhibition assay for HIF-P4H-1 using increasing concentrations of glutarate. **d**, HIF-PH activity luciferase reporter assay in mouse embryonic fibroblasts (MEFs) treated for 16 h with increasing concentrations of DEG. Data points are the mean ± s.e.m. *n* = 9. **e**, HIF-PH activity luciferase reporter assay in MEFs treated for 16 h with DEG and dimethyl αKG (DMαKG). Two-way ANOVA; *n* = 3. RLU, relative light unit. **f**, Representative western blot and log_2_ fold change in protein expression of specific histone 3 (H3) methylation sites in human CD8^+^ T cells treated for 7 days with or without DEG 500 µM. Each dot represents one human donor normalized to total H3 and relative to untreated control (dashed black line). Two-sided, one-sample *t*-test; H3K4me3, *n* = 6; H3K9me2, *n* = 11; H3K9me3, *n* = 11; H3K27me3, *n* = 10. **g**, Representative western blots and log_2_ fold change protein expression of H3K27me3 in human CD8^+^ T cells treated with or without 500 µM DEG or 500 µM αKG for 7 days. Ordinary one-way ANOVA; DEG, *n* = 10; αKG, *n* = 3; αKG + DEG, *n* = 3. **h**, Representative western blots and log_2_ fold change protein expression of H3K9me3 in human CD8^+^ T cells treated with or without 500 µM DEG or 500 µM αKG for 7 days. Ordinary one-way ANOVA; *n* = 3–5. DEG, *n* = 11; αKG, *n* = 3; αKG + DEG, *n* = 3. **i**, Cell-free enzymatic inhibition assay for KDM4C using increasing concentrations of glutarate. **j**, Cell-free enzymatic inhibition assay for KDM6A using increasing concentrations of glutarate. Cell-free enzymatic assay graphs showing Michaelis–Menten line of best fit of at least three independent experiments. All scatter plots show the median + 95% CI, where each dot represents one donor as indicated. *P < 0.05, **P < 0.01, ***P < 0.001, ****P < 0.0001. Source data

In cell-free enzymatic activity assays, glutarate inhibited recombinant TET2 with a half maximal inhibitory concentration (IC_50_) of 1.5 mM (Fig. 2a, left). This inhibition is competitive with αKG, as indicated by reduced inhibition with increased levels of αKG (Fig. 2a, right). Competitive inhibition of TET2 correlated with an observed decrease in 5hmC (the reaction product of TET2) levels in a dose-dependent manner in human CD8^+^ T cells cultured with DEG for 7 days (Fig. 2b). DEG did not inhibit TET2 in cell-free assays (Extended Data Fig. 2b), providing further evidence that our observed results are due to free glutarate.

Glutarate also inhibited the HIF-proyl4hydroxylase-1 (HIF-P4H-1) enzyme in vitro, with an IC_50_ of 2 mM in a cell-free assay (Fig. 2c). To test HIF-P4H inhibition via DEG treatment in a cellular system, we used a luciferase reporter assay driven by a Gal4 response element (GRE-luc), pFLAG-Gal4-mHIF-1α N-terminal transcription activation domain (NTAD)^35^. The NTAD contains proline residues that are hydroxylated by HIF-P4H enzymes to target the protein for degradation; thus, luciferase expression in this assay is controlled by HIF-P4H hydroxylation activity against the pFLAG-Gal4-mHIF-1α NTAD. We found that DEG can inhibit cellular HIF-P4H activity in this model system (Fig. 2d). HIF-P4H-1 inhibition by glutarate was reduced when increasing concentrations of αKG were added to the culture (Fig. 2e), indicating that, as with TET2 inhibition, glutarate inhibits HIF-P4H-1 in an αKG-competitive manner. This inhibition of HIF-P4H-1 was correlated with increased expression of several HIF target genes in DEG-treated CD8^+^ T cells for 7 days (Extended Data Fig. 2c).

To determine if glutarate also inhibits KDM enzymes, we cultured CD8^+^ T cells with DEG for 7 days and determined the methylation status of a number of histone 3 (H3) lysine residues (Fig. 2f). DEG treatment of CD8^+^ T cells increased dimethylation and trimethylation of H3K9 and trimethylation of H3K27 (Fig. 2f). This increase in H3K9 and H3K27 trimethylation was abrogated by the addition of equimolar amounts of αKG (Figs. 2g,h), while H3K9me2 levels were not reduced with the addition of αKG (Extended Data Fig. 2d). As KDM4C and KDM6A are responsible for the demethylation of H3K9me3 and H3K27me3, respectively, we performed cell-free enzymatic activity assays with these enzymes and found that glutarate inhibited KDM4C with an IC_50_ of 1 mM and KDM6A with an IC_50_ of 0.6 mM (Figs. 2i,j). As expected, DEG alone was not capable of inhibiting KDM4C activity in a cell-free assay (Extended Data Fig. 2e).

These data indicate that glutarate is an inhibitor of at least three specific αKGDDs, namely, the TET2, HIF-P4H-1 and KDM4C/6A enzymes. This inhibition correlates with multiple changes to cellular gene expression and epigenetic profiles, including specific HIF targets, increased histone methylation and decreased DNA demethylation.

### Glutarylation of PDH disrupts lipoylation

**Fig. 3:**
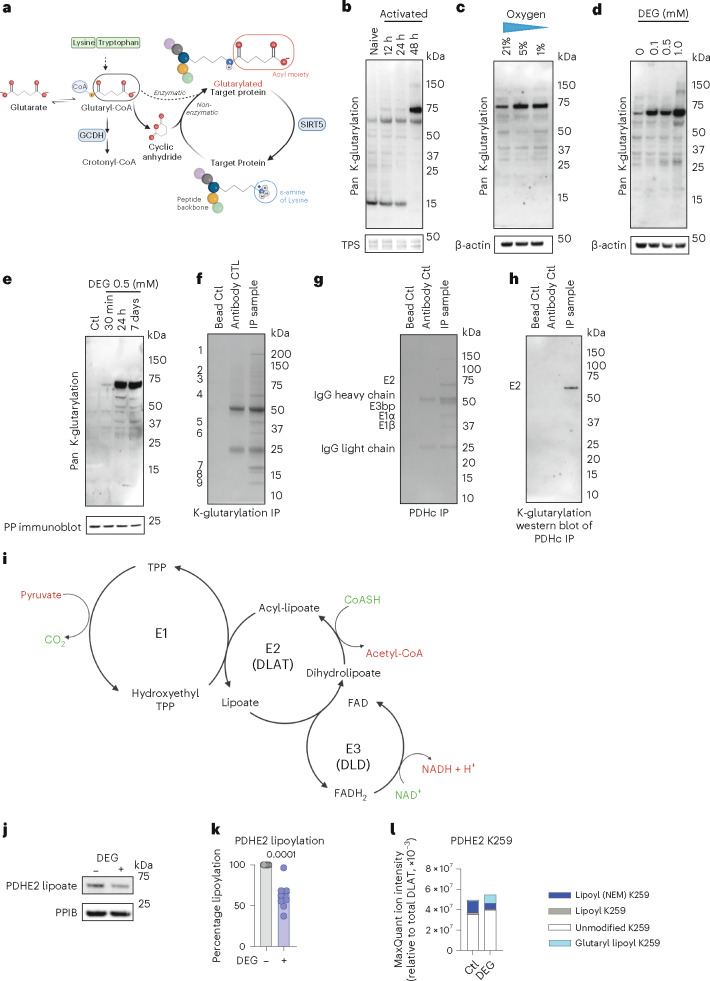
Glutarylation of PDH disrupts lipoylation. **a**, Model of protein lysine glutarylation (adapted from BioRender.com)*.* **b**, Representative western blot of naive and activated human CD8^+^ T cells. *n* = 3. TPS, total protein stain. **c**, Representative western blot of activated human CD8^+^ T cells cultured at different oxygen tensions (21%, 5% or 1%). *n* = 3. **d**, Representative western blot of human CD8^+^ T cells cultured with increasing concentrations of DEG for 7 days. *n* = 4. **e**, Representative western blot of activated human CD8^+^ T cells cultured with 500 µM DEG for several different time lengths as indicated. All samples were collected 7 days after activation. *n* = 5. **f**, Representative Coomassie brilliant blue staining of proteins immunoprecipitated with a pan-K-glutarylation antibody and separated by SDS–polyacrylamide gel electrophoresis (PAGE). A total of 30 × 10^6^ mouse CD8^+^ T cells, 7 days after activation, were used. *n* = 3. **g**, Representative Coomassie brilliant staining of immunoprecipitated PDHc separated by SDS–PAGE. A total of 30 × 10^6^ mouse CD8^+^ T cells, 7 days after activation, were used. *n* = 3. **h**, Representative K-glutarylation western blot of immunoprecipitated PDHc as described in Fig. 4g. *n* = 3. **i**, Schematic of the reactions catalysed by the individual subunits of the PDHc (adapted from BioRender.com). **j**, Representative western blot of PDHE2 lipoate levels in human CD8^+^ T cells treated with 500 µM DEG for 24 h. *n* = 12. **k**, Percentage lipoylation calculated from Fig. 4k, where untreated control represents 100% lipoylation. Two-tailed, paired *t*-test; *n* = 12. **l**, Quantified proteomic analysis of select PTMs on PDHE2 K259, relative to total PDHE2, using HeLa cells treated with 500 µM DEG for 24 h. Source data

Glutarylation, a recently discovered post-translational modification (PTM), uses glutarate in its CoA form, glutaryl-CoA, as a substrate. The reaction can occur both enzymatically and non-enzymatically, while deglutarylation is thought to be mediated by SIRT5 (refs. ^36–38^) (Fig. 3a). Recently, studies of mouse liver and brain showed that glutarylation can modify a range of proteins^36,39^. Given our observation of fluctuating levels of glutarate in CD8^+^ T cells (Fig. 1f,i), we wished to determine whether glutarylation also takes place in CD8^+^ T cells.

**Extended Data Fig. 3:**
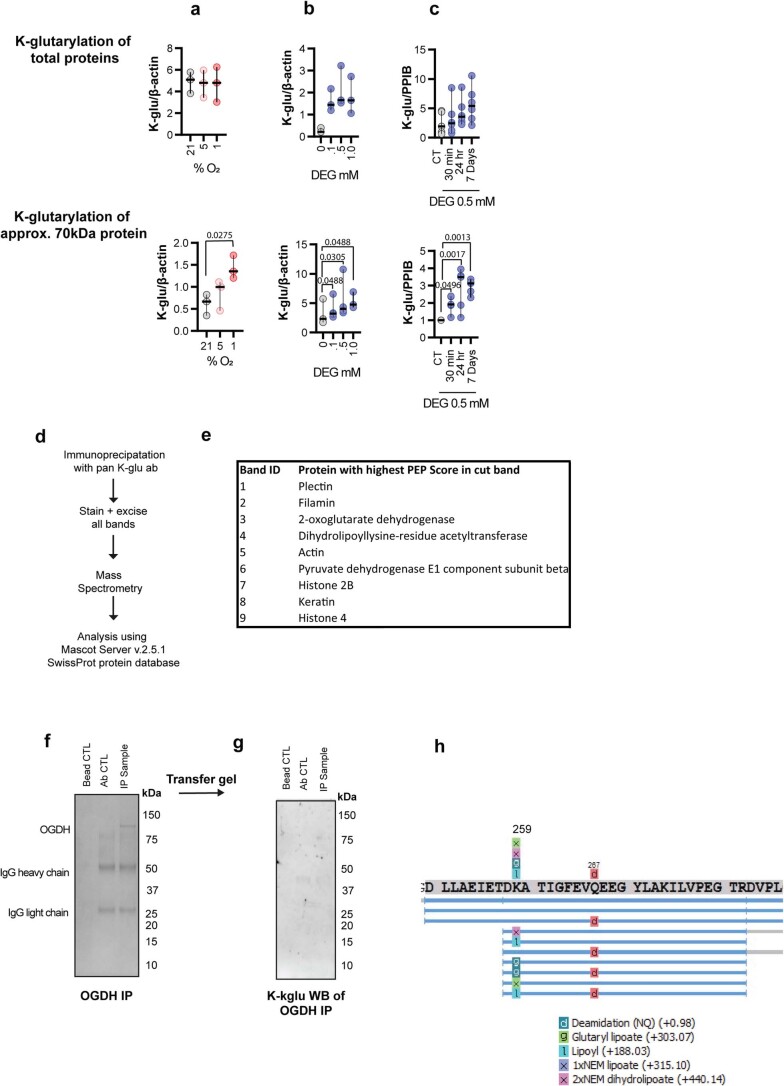
Glutarylation of pyruvate dehydrogenase disrupts lipoylation. **a**, Quantified total protein lysine-glutarylation or lysine-glutarylation of 67-70 kDa protein, as determined by western blot analysis and relative to β-actin, of human CD8^+^ T cells which were cultured at different oxygen tensions for 24 h prior to harvest (as in described in Fig. 4c). Ordinary one-way ANOVA; n = 3. **b**, Quantified total protein lysine-glutarylation or lysine-glutarylation of 67-70 kDa protein, as determined by western blot analysis and relative to β-actin, of cells treated with increasing concentrations of DEG for 7 days (as described in Fig. 4d). Ordinary one-way ANOVA; n = 3. **c**, Quantified total protein lysine-glutarylation or lysine-glutarylation of 67-70 kDa protein, as determined by western blot analysis and relative to PPIB, of human CD8^+^ T cells 7 days post activation. Cells were treated with DEG 500 µM for different time lengths as indication and harvested at the same time 7 days post activation (as described in Fig. 4e). Ordinary one-way ANOVA; n = 5. **d**, Model of lysine-glutarylated protein isolation and identification by mass spectrometry. Lysine-glutarylation immunoprecipitation was performed on 30×10^6^ mouse CD8^+^ T cells, 7 days post activation. Following protein separation by SDS-PAGE, gel was stained with Coomassie blue and stained bands were excised, gel was digested and mass spectrometry was performed. Data was analysed using the Mascot Server v.2.3.1 and the SwissProt protein database. 3 independent experiments and analysis performed. **e**, Top hits for each excised band from mass spectrometry described in Fig. 4g. **f**, Representative immunoprecipitation of Oxoglutarate dehydrogenase (OGDH) complex isolated from 30×10^6^ mouse CD8^+^ T cells by immunoprecipitation. N = 3. **g**, Representative K-glutarylation western blot of immunoprecipitated OGDH complex as described in Extended Data Fig. 4f. N = 3. **h**, Schematic illustrating PEAKS PTM K259 on PDHE2 (selected modifications). All scatter plots show median and 95% CI where each dot represents one human donor. Source data

We determined this first by undertaking whole-protein lysate western blot analyses with a pan-lysine glutarylation (K-glutarylation) antibody^36,38^. We found that protein glutarylation is clearly detectable in CD8^+^ T cells (Fig. 3b). We also found that glutarylation patterns change strikingly after CD8^+^ T cell activation (Fig. 3b). Glutarylation patterns were also altered when cells were exposed to different oxygen tensions (Fig. 3c and Extended Data Fig. 3a) and when treated with DEG (Fig. 3d and Extended Data Fig. 3b). Interestingly, shifts in glutarylation could be detected after just 30 min of culture with DEG (Fig. 3e and Extended Data Fig. 3c). Since, as shown in Extended Data Fig. 1g, DEG is converted within 15 min to glutarate intracellularly, it is likely that the DEG-induced glutarylation observed after 30 min of DEG treatment is a direct result of increases in intracellular glutarate.

In these western blot assays we observed a relatively small number of proteins with marked changes in glutarylation caused by T cell activation (Fig. 3b–e). One notable protein in this assay was a protein of approximately 70 kDa. This protein was also the moiety most affected by hypoxia and by exposure of T cells to DEG (Extended Data Fig. 3a–c). To establish the identity of this and other glutarylated proteins in CD8^+^ T cells, we performed an immunoprecipitation (IP) assay with a K-glutarylation antibody (Fig. 3f). We then excised the observed bands and used mass spectrometry in conjunction with the SwissProt protein database to identify them (Extended Data Fig. 3d). Proteins with the highest posterior error probability (PEP) score in each band are listed in Extended Data Fig. 3e.

Three non-cytoskeletal/histone proteins were positively identified; (1) 2-oxoglutarate dehydrogenase (OGDH); (2) dihydrolipoyl lysine residue acetyltransferase (DLAT) (also known as the E2 subunit of the PDHc (PDHE2)); and (3) PDHE1 component subunit beta (PDHE1β) (Extended Data Fig. 3e). Glutarylation targets were confirmed by reverse IP, in which an IP with the target antibody was performed and then the resulting western blot was probed with the K-glutarylation antibody.

IP and subsequent western blot analysis of the PDHc (which includes PDHE2 and PDHE1β) revealed that it was the PDHE2 subunit alone that in our assays was clearly and detectably glutarylated (Fig. 3g,h). IP and subsequent western blot analysis with the K-glutarylation antibody revealed that OGDH is not detectably glutarylated in this system (Extended Data Fig. 3f,g). Thus, the PDHE2 subunit (67 kDa) was the only glutarylation target identified both by mass spectrometry and subsequently confirmed by reverse IP.

PDHc catalyses the conversion of pyruvate to acetyl-CoA and thus links glycolysis and the tricarboxylic acid (TCA) cycle^40^. PDHc consists of multiple copies of three catalytic enzymes: PDH (E1); dihydrolipoamide acetyltransferase (PDHE2); and dihydrolipoamide dehydrogenase (DLD) (E3) (Fig. 3i). The E2 subunit is the functional core of the PDHc and provides catalytic activity via cyclical reduction and oxidation of its lipoyl domains, channelling substrates between individual enzymes’ active sites in the PDHc. The multidomain E2 subunit consists of the C-terminal catalytic domain, the peripheral subunit-binding domain and the N-terminal ends of 1–3 lipoyl domains^41–44^. The lipoyl domains contain one lipoic acid covalently attached to a lysine residue; this lipoic acid is essential for PDHc catalytic activity^45^.

Following the observation that lysine residues of PDHE2 can be glutarylated, we sought to determine if lysine glutarylation was disrupting lipoic acid conjugation to PDHE2 lysine residues, thereby disrupting normal PDHc functioning. We treated human CD8^+^ T cells with DEG and observed reduced PDHE2 lipoate level (Fig. 3j,k). Proteomic analysis of PDHE2 immunoprecipitated from HeLa cells revealed that DEG treatment reduced the levels of lipoyl K259, while increasing glutaryl-lipoyl K259 (Fig. 3l and Extended Data Fig. 3h). In addition to the observed glutarylation site on K259, two additional PDHE2 lysine residues were found by proteomics to have glutaryl groups attached, that is, K396 and K482.

These data illustrate that glutarylation occurs in CD8^+^ T cells, that this glutarylation status changes after activation, and identifies PDHE2 as a glutarylation target, while providing evidence that glutarylation disrupts normal PDHc lipoylation, potentially altering the catalytic activity of the complex.

### Glutarate modulates metabolic state

**Fig. 4:**
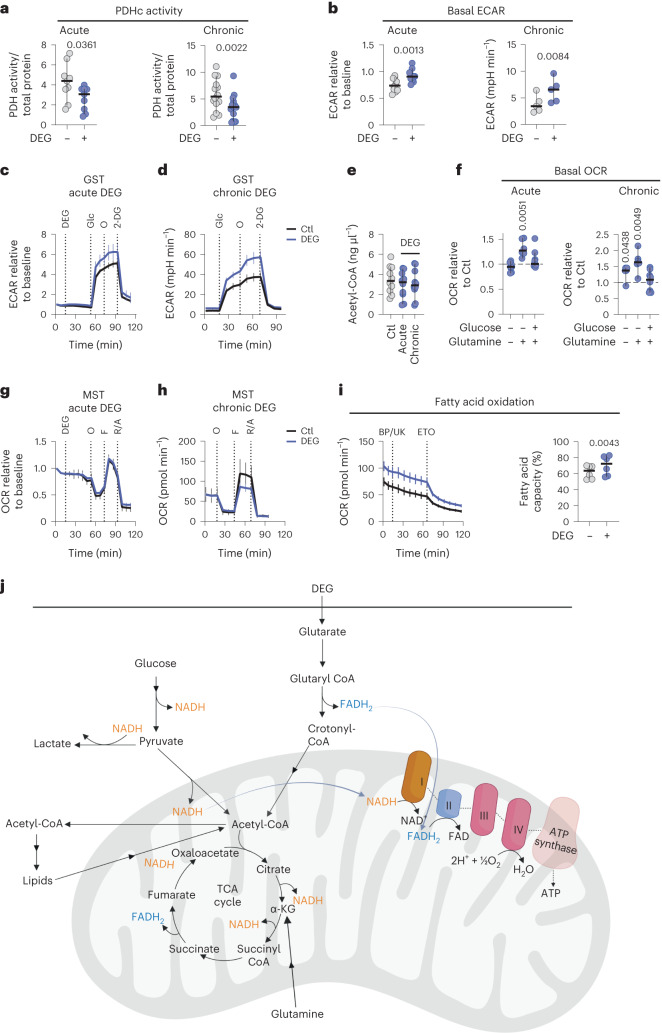
Glutarate modulates mitochondrial function by inhibiting PDH. **a**, PDHc activity of CD8^+^ T cells treated with DEG for 30 min (acute) or 7 days (chronic), normalized to total protein concentration. Two-tailed, paired *t*-test; acute, *n* = 9; chronic, *n* = 14. **b**, Basal ECAR in CD8^+^ T cells as determined by Seahorse analysis after 30-min DEG treatment via Seahorse injection (acute, left) or after 7 days of in vitro culture with DEG (chronic, right). Acutely treated cells normalized to ECAR levels before DEG/Ctl injection. Two-tailed, paired *t*-test; acute, *n* = 10; chronic, *n* = 5. **c**, Seahorse analysis of ECAR levels in CD8^+^ T cells 7 days after activation. *n* = 10. **d**, Seahorse analysis of ECAR measurements during a standard GST of CD8^+^ T cells treated with or without DEG for 7 days. *n* = 6. **e**, Acetyl-CoA levels in CD8^+^ T cells treated with DEG for 30 min (acute) or 7 days (chronic). Repeated measures one-way ANOVA; *n* = 10. **f**, Basal OCR levels in CD8^+^ T cells as determined by Seahorse analysis after 30-min DEG treatment via Seahorse injection. During the assay, cells were plated in XF medium with or without glucose and glutamine as indicated. OCR is represented as the fold change relative to untreated control. Two-tailed, one-sample *t*-test; acute, + glucose, − glutamine, *n* = 8; acute, − glucose, + glutamine, *n* = 6; acute, + glucose, + glutamine, *n* = 10; chronic, − glucose, − glutamine, *n* = 5; chronic, − glucose, + glutamine, *n* = 6; chronic, + glucose, + glutamine, *n* = 8. **g**, Seahorse analysis of OCR in CD8^+^ T cells (7 days after activation). *n* = 10. **h**, Seahorse analysis of OCR measurements during a standard GST of CD8^+^ T cells treated with or without DEG for 7 days. *n* = 6. **i**, Fatty acid oxidation capacity as determined by OCR measurements during a mitochondrial fuel flex test of CD8^+^ T cells treated with or without DEG for 7 days as described in Extended Data Fig. 4i. Calculated percentage of fatty acid capacity (right): two-tailed, paired *t*-test; *n* = 6. **j**, Schematic illustrating the contribution of glutarate, glucose, lipids and glutamine to cellular metabolism (adapted from BioRender.com). All experiments shown used 500 µM DEG. All scatter plots show the median + 95% CI, where each dot represents one human. The ECAR and OCR time course graphs show the mean and error with the 95% CI.

**Extended Data Fig. 4:**
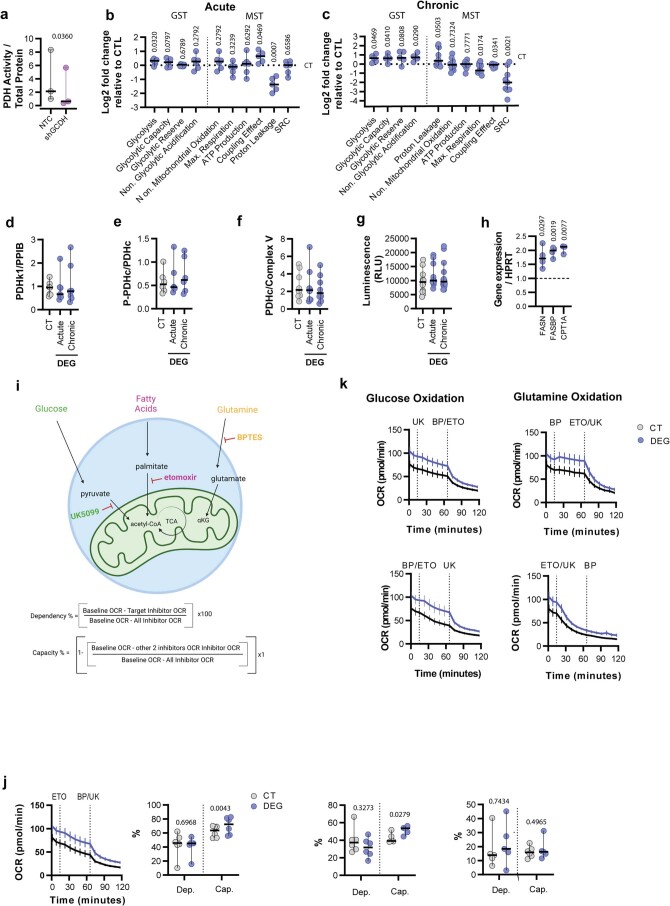
Glutarate modulates mitochondrial function by inhibiting pyruvate dehydrogenase. **a**, PDHc activity of CD8^+^ T cells transduced with a NTC or shGCDH normalised to total protein. Two-tailed unpaired t-test; n = 3. **b-c**, Quantitative analysis of MST and GST following acute (30 min) (left) and chronic (7 days) (right) DEG exposure, as described in Fig. 4c-d and Fig. 4h-i, shown as log_2_ FC relative to untreated control (black dotted line). Two-tailed one sample t-test; Acute GST n = 6; Acute MST n = 6; Chronic GST n = 5, Chronic MST n = 10. **d**, Protein expression of PDHK1 as determined by western blot analysis. Ordinary one-way ANOVA; n = 7. **e**, Protein expression of phosphorylated PDHE1α (ser239) as determined by western blot analysis. Ordinary one-way ANOVA; n = 7. **f**, Protein expression of PDHc as determined by western blot analysis. Ordinary one-way ANOVA; n = 8. **g**, Glucose uptake by human CD8^+^ T cells treated with DEG. Data represented as relative light units (RLU). Mixed-effects analysis; n = 9 (acute), n = 14 (chronic). **h**, qPCR analysis of CD8^+^ T cells treated with DEG for 7 days. Each dot represents one human donor relative to untreated donor control (dotted black line), normalised to HPRT. Two-tailed one sample t-test; FASN n = 4, FASBP n = 4; CPT1A n = 3. **i**, Model of mitochondrial fuel flexibility tests and illustration of calculations used to determine mitochondrial fuel oxidative capacity and dependency. *(Adapted from BioRender.com)* **j**, Fatty acid oxidation dependency as determined by OCR measurements during a mitochondrial fuel flex test of CD8^+^ T cells treated with +/- DEG 500 µM for 7 days as described in Extended Data Fig. 4i. Calculated percentage fatty acid dependency (right): Two-tailed paired t-test; n = 5. **k**, OCR measurements and percentage fuel oxidative capacity (cap) or dependency (dep) during a mitochondrial fuel flex test of CD8^+^ T cells treated with +/- DEG 500 µM for 7 days, as described in Extended Data Fig. 4i. Calculated percentage fuel capacity and dependency (bottom): Two-tailed paired t-test; Glucose oxidation dependency n = 6; Glucose oxidation capacity n = 5; Glutamine oxidation dependency n = 5; Glutamine oxidation capacity n = 6. All experiment describes used 500 µM DEG. All scatter plots show median and 95% CI where each dot represents one human donor. OCR time course graphs show Mean and Error with 95% CI. Source data

PDHc links glycolysis to the TCA cycle as noted above; thus, we hypothesized that glutarylation might decrease PDHc enzymatic activity, with a resultant increased glycolytic rate. To test this, we performed a direct enzymatic activity assay of the PDHc on CD8^+^ T cell lysates and found that both acute (30 min) and chronic (seven day) exposure to DEG resulted in decreased PDHc activity (Fig. 4a). Reduced PDHc activity was also observed in CD8^+^ T cells with partially silenced GCDH, and thus increased endogenous glutarate levels (Extended Data Fig. 4a). As expected, this reduction in PDHc activity resulted in increases in the basal extracellular acidification rate (ECAR) (Fig. 4b), as pyruvate was metabolized by the lactate dehydrogenase enzyme to form lactate.

To explore further the metabolic consequences of this observed glutarate-induced reduction in PDHc activity, we performed a glycolysis stress test (GST). Acute effects were determined by treating CD8^+^ T cells with DEG for 30 min while plated in the Seahorse XF Analyzer. Chronic effects were determined by performing a standard GST in CD8^+^ T cells that had been cultured with DEG for 7 days before assay. Both acute and chronic exposure to DEG resulted in an increased glycolytic rate (Fig. 4c,d and Extended Data Fig. 4b,c).

Glycolysis is highly regulated by the HIF transcription factor, and because, as shown above, glutarate is a competitive inhibitor of HIF-P4H-1 (Fig. 2c–e) and thus can induce the transcription of HIF target genes (Extended Data Fig. 2c), we next sought to determine the relative contribution of glutarate-induced HIF activity to the observed metabolic changes we observed: reduced PDHc activity and increased glycolysis. Phosphorylation of PDHc by pyruvate dehydrogenase kinase (PDHK) is a known regulator of PDHc activity, and although DEG treatment of CD8^+^ T cells increased the mRNA levels of PDHK1 (Extended Data Fig. 2c), PDHK1 proteins levels were not significantly altered after DEG treatment (Extended Data Fig. 4d). Importantly we did not observe any changes in PDHc phosphorylation levels after DEG treatment (Extended Data Fig. 4e). Additionally, DEG treatment did not alter the protein expression of the PDHc (Extended Data Fig. 4f).

The glucose transporter GLUT1 is also a HIF-regulated gene, and so we also determined the rate of glucose uptake in cells treated with DEG. Again, we observed no changes, which is consistent with no HIF-induced functional effects (Extended Data Fig. 4g). Although as shown above, glutarate is a competitive inhibitor of HIF-P4H-1 (Fig. 2c–e), a more extended or higher exposure to glutarate than those found under our experimental conditions may be required to elicit a functional HIF response. Taken together, these data suggest that the key factor in the alteration of glycolytic rate in DEG-treated cells is glutarylation of PDHE2, and that it is this PTM of PDHE2 that is responsible for both altered PDHc activities in DEG-treated cells and the increases in ECAR and glycolytic rate observed after treatment.

Despite reduced PDHc activity, acetyl-CoA levels were not lower after in vitro treatment with DEG (Fig. 4e), indicating potential compensation from alternative pathways. Additionally, mitochondrial respiration, as characterized by cellular basal oxygen consumption rates (OCRs), was either maintained or increased after DEG exposure (Fig. 4f). Glutarate in its CoA form, glutaryl-CoA, can form crotonyl-CoA before being further metabolized to acetyl-CoA; so while glutaryl-CoA may reduce acetyl-CoA generation from pyruvate via glutarylation of PDHE2, acetyl-CoA levels, and thus mitochondrial oxidation, can be maintained as glutarate and used as a direct fuel source (Fig. 4j). During a standard mitochondrial stress test (MST), the addition of carbonyl cyanide-*p*-trifluoromethoxyphenylhydrazone (FCCP), the mitochondrial uncoupling agent, did not have an effect on OCR in cells treated acutely with DEG (Fig. 4g), while cells that had been treated for 7 days with DEG before assay had a reduced maximal OCR (Fig. 4h), indicating that although glutarate can maintain OCR, it is not as efficient as glucose in facilitating mitochondrial oxidation.

To better understand the consequences of providing glutarate as a fuel for mitochondrial oxidation, we measured cellular oxygen consumption while inhibiting, in different combinations, fatty acid, glucose and glutamine oxidation, the three main fuel sources for mitochondrial oxidation (Extended Data Fig. 4i). This was achieved via injection of etomoxir, UK5099 and Bis-2-(5-phenylacetamido-1,3,4-thiadiazol-2-yl)ethyl sulphide (BPTES), respectively, in cells plated in a Seahorse XF Analyzer. We found that while DEG-treated cells are not more dependent on any of these pathways (Extended Data Fig. 4j,k), treated cells do have an increased capacity to oxidize both fatty acids (Fig. 4i) and glucose (Extended Data Fig. 4k) when other fuel sources are limited. Additionally, these cells have increased levels of fatty acid synthase (*FASN*), fatty acid binding protein (*FABP*) and carnitine palmitoyltransferase 1A (*CPT1A*) gene expression (Extended Data Fig. 4h), suggesting that acetyl-CoA formed from glutarate breakdown may be used to generate fats via fatty acid synthesis, and that these fats can be used when there is a depletion of other nutrients.

These data illustrate that glutarate levels can modulate CD8^+^ T cell metabolism.

### Glutarate treatment in vivo acts to reduce tumour growth

The metabolism of CD8^+^ T cells is a critical aspect of their effector function^46^. Given our observations, in addition to the recent observation that PDHc inhibition can increase CD8^+^ T cell cytotoxicity and increase immunotherapeutic efficacy^47^, and the ongoing clinical trials using PDHc inhibitors for several cancers^48,49^, we sought to determine the effects of DEG in tumour immunotherapy models.

**Fig. 5:**
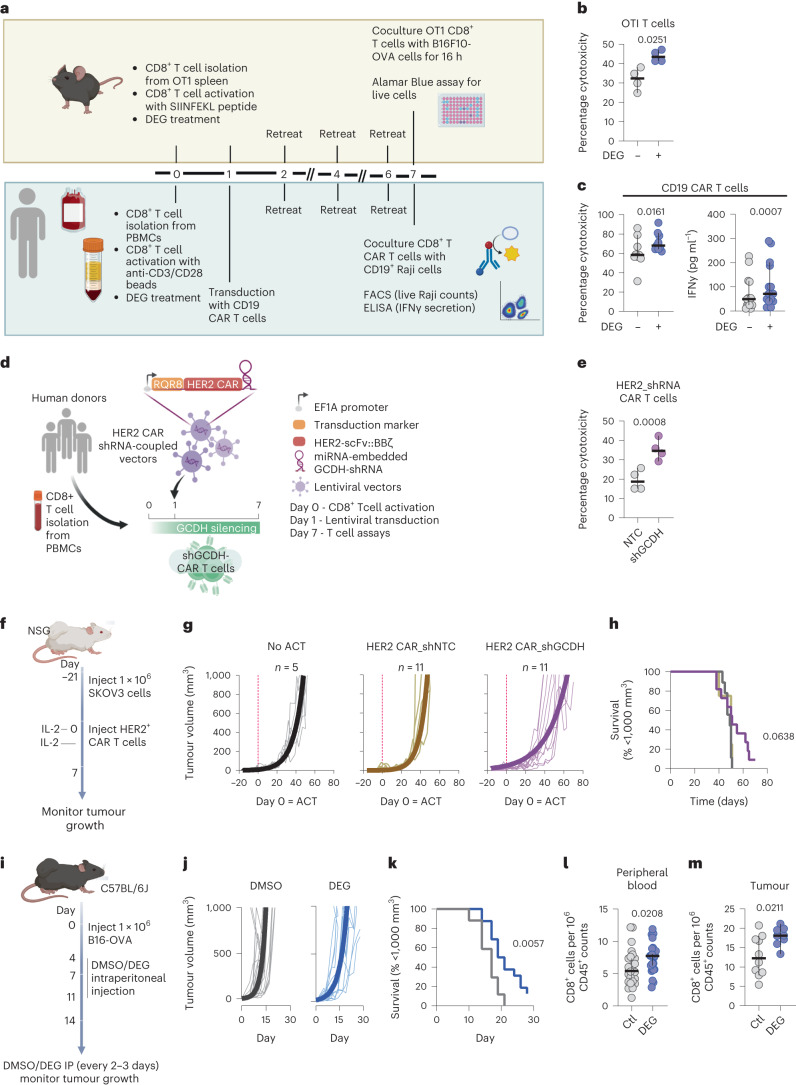
Glutarate reduces tumour growth and increases CD8^+^ T cell numbers and tumour infiltration. **a**, Model of in vitro cytotoxicity assays; 500 µM DEG was used (adapted from BioRender.com)*.* ELISA, enzyme-linked immunosorbent assay; FACS, fluorescence-activated cell sorting. **b**, Percentage cytotoxicity of OT1 cells after coculture with B16F10-OVA cells as described in **a**. Two-tailed, paired *t*-test; *n* = 4. **c**, Percentage cytotoxicity (left) and IFNγ expression (right) of CD19 CAR T cells after coculture with CD19^+^ Raji cells as described in **a**. Two-tailed, paired *t*-test; cytotoxicity, *n* = 9; IFNγ, *n* = 14. **d**, Model of HER2 CAR T shRNA cell generation (adapted from BioRender.com). **e**, Percentage cytotoxicity of HER2 CAR T cells with embedded shGCDH or shNTC as determined by Alamar Blue assay after coculture with HER2-expressing SKOV3 cells. Two-tailed, paired *t*-test; *n* = 4. **f**, Adoptive cell therapy (ACT) model with CAR T cells. Tumour growth was monitored every 2–3 days until day 70. **g**, Tumour growth data. The thin lines represent tumour growth from individual mice and the thick lines represent an exponential (Malthusian) growth curve. **h**, Survival curves using 1,000 mm^3^ tumour volume as the threshold. log-rank (Mantel–Cox) test. **i**, Tumour growth model; 1.0 × 10^6^ B16F10-OVA cells were injected subcutaneously into C57BL/6J mice. From day 4 after tumour inoculation, mice were injected interperitoneally with 10 mg kg^−1^ DEG or dimethyl sulfoxide (DMSO) Ctl every 2–3 days. On day 14 after tumour inoculation, peripheral blood, tumour, spleen and tumour-draining lymph node from some mice were processed to single-cell suspensions and analysed by flow cytometry. Tumour growth was monitored until day 30. **j**, Tumour growth data. The thin lines represent tumour growth from individual mice and the thick lines represent an exponential (Malthusian) growth curve. **k**, Survival curves using 1,000 mm^3^ tumour volume as the threshold. log-rank (Mantel–Cox) test; *n* = 14. **l**, Frequency of CD8^+^ T cells in peripheral blood 14 days after tumour inoculation. Two-tailed *t*-test; *n* = 25. **m**, Frequency of CD8^+^ T cells in the tumours 14 days after tumour inoculation. Two-tailed, *t*-test; Ctl, *n* = 10; DEG, *n* = 8. All scatter plots show the median and 95% CI, where each dot represents one donor (human or murine as indicated).

**Extended Data Fig. 5:**
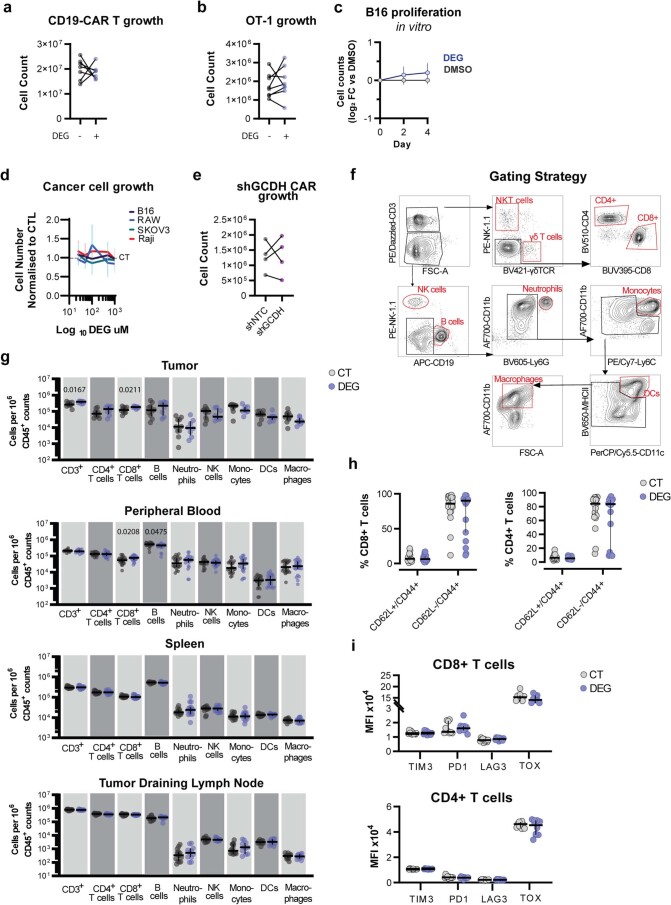
Glutarate reduces tumor growth and increases T cell numbers and tumor infiltration. **a**, Cell count of human CAR-T cells treated +/- 500 µM for 7 days. Two-tailed paired t-test; n = 6. **b**, Cell count of murine OT1 CD8^+^ T cells treated +/- 500 µM for 7 days. Two-tailed paired t-test; n = 6. **c**, *In vitro* B16F10-OVA cell growth +/- DEG 500 µM as determined by cell counting and represented as log_2_ FC. One way ANOVA; n = 3. **d**, Cell count of different cancer cell lines treated with increasing concentrations of DEG for 72 h. Cell count relative to appropriate untreated control. One way ANOVA; n = 3. **e**, Cell count of human HER2-CAR-T_shRNA cell numbers 7 days post transduction. Two-tailed paired t-test; n = 4. **f**, Gating strategy used for analysis of Fig. 5-m and Extended Data Fig. 5g. **g**, Frequency of immune cell types in peripheral blood, tumor, spleen, and tumor draining lymph nodes, 14 days post tumor inoculation, represented as log_2_ FC relative to CD45^+^ count. Two-tailed unpaired t-test; Tumor CT n = 10; Tumor DEG n = 8; Peripheral Blood CT + DEG n = 15; Spleen CT + DEG n = 15; Tumor Draining Lymph Nodes CT n = 15; Tumor Draining Lymph Nodes DEG n = 15. **h**, Percentage of CD8^+^/CD4^+^ T cells expressing CD44/CD62L in tumors 14 days post tumor inoculation. Two-tailed unpaired t-test; Ct n = 20; DEG n = 18. **i**, MFI of TIM3, PD1, LAG3 and TOX on CD8^+^/CD4^+^ T cells in tumor s, 14 days post tumor inoculation. Two-tailed unpaired t-test; n = 10. Graph shows median + 95% CI where each dot represents one murine or human donor as indicated. *p < 0.05, **p < 0.01.

To test this, after 7 days of culture in DEG-containing medium, antigen-specific cytotoxicity was first determined in both a human CAR T cell system derived from human donor CD8^+^ T cells, and a murine system using transgenic OT1 CD8^+^ T cells (Fig. 5a). After 7 days of culture with DEG, murine OT1 CD8^+^ T cells were more proficient at killing ovalbumin (OVA)-expressing B16F10-OVA cancer cells (Fig. 5b), while DEG-treated human CD19^+^ CAR T cells secreted more interferon-γ (IFNγ) and exhibited increased cytotoxicity against CD19^+^ Raji cells (Fig. 5c). DEG treatment did not affect the growth of either the human CD19^+^ CAR T cells or the murine OT1 T cells (Extended Data Fig. 5a,b) or cause any toxicity in the tumour cells themselves (Extended Data Fig. 5c,d).

To determine whether increased endogenous glutarate levels in CD8^+^ T cells also increases the cytotoxic efficacy of CD8^+^ T cells, we created a human HER2 CAR T system containing an miRNA-embedded shRNA against GCDH (HER2 CAR T_shGCDH) (Fig. 5d). HER2 CAR T cells with a non-targeted control (NTC) (HER2 CAR T_shNTC) were used as a control. CD8^+^ T cells expressing HER2 CAR T_shGCDH had significantly increased cytotoxicity against target HER2^+^ SKOV3 ovarian cancer cells when compared to control HER2 CAR T_shNTC cells (Fig. 5e). Cell growth was not affected in shGCDH-expressing cells (Extended Data Fig. 5e).

We next performed in vivo tumour growth experiments in which mice with HER2^+^ SKOV3 tumours were treated with HER2 CAR T_shGCDH cells (Fig. 5f). Mice treated with HER2 CAR T_shGCDH cells had reduced rates of tumour growth in 6 of 11 mice compared to mice treated with HER2 CAR T_shNTC cells (Fig. 5g). However, we did not observe a difference in the overall survival of mice (Fig. 5h).

We next wished to determine whether direct administration of DEG to tumour-bearing mice would result in a slowing of tumour growth. DEG is non-toxic and is in fact approved by the European Union for use as a food additive^50^. To determine whether it could be used therapeutically, we carried out an experiment in which mice with B16F10-OVA melanoma tumours were injected intraperitoneally with DEG every 48–72 h (Fig. 5i). This treatment regimen significantly slowed tumour growth and significantly increased survival of DEG-treated mice (Fig. 5j,k).

To determine the mechanisms underlying the reduced tumour growth we observed in vivo after DEG treatment, we again undertook treatment of B16F10-OVA tumour-bearing mice with DEG every 48–72 h as described above. Ten days after the start of DEG treatment, we collected peripheral blood, tumours, spleens and tumour-draining lymph nodes from the experimental animals. Single-cell suspensions were generated from each sample and the quantity of several immune cell populations was determined (Extended Data Fig. 5f,g). CD3^+^ T cells, CD4^+^ T cells, CD8^+^ T cells, B cells, neutrophils, natural killer cells, monocytes, dendritic cells and macrophages were assayed for, and this analysis revealed a significant increase in both peripheral blood CD8^+^ T cells and tumour-infiltrating CD8^+^ T cells in DEG-treated animals relative to controls (Fig. 5l,m). No changes in other immune populations were observed, apart from a slight decrease in circulating B cells (Extended Data Fig. 5g). The CD8^+^ T cell phenotypic changes observed with ex vivo treatment with DEG (Fig. 1) are not observed in tumour-infiltrating CD8^+^ T cells in DEG-treated mice (Extended Data Fig. 5). Analysis of tumour-infiltrating lymphocytes revealed no changes in the expression of memory-associated markers (CD62L and CD44) or exhaustion-associated markers (TIM3, PD1, LAG3, TOX) in CD4^+^ or CD8^+^ cells (Extended Data Fig. 5h,i). Phenotypic analysis of circulating CD8^+^ T cells in DEG-treated mice was not performed in this study. Future studies will perform detailed analysis of both circulating and tumour-infiltrating CD8^+^ T cells in tumour-bearing mice treated with DEG to fully elucidate the in vivo effects of DEG treatment.

These data illustrate that glutarate can enhance CD8^+^ T cell cytotoxicity, and that in vivo treatment of tumour-bearing mice with an esterified form of glutarate can reduce tumour growth and increase animal survival, highlighting the use of this immunometabolite as a potential cancer therapy.

## Discussion

The intrinsic link between metabolism and cellular function was first highlighted by the discovery of mutations in genes encoding metabolic enzymes. Mutations in fumarate hydratase, succinate dehydrogenase and isocitrate dehydrogenase, all of which are key enzymes of the TCA cycle, lead to the accumulation of fumarate, succinate and 2HG, respectively. At high levels, each of these metabolites can act as disease-driving signalling molecules.

Mutations in the GCDH enzyme can also be pathological and detrimental to normal development^26^. There is also at least one report indicating that an unusual occurrence of glioblastoma has been found in patients with mutations in *GCDH*^51^. However, to date, the functions of glutarate have been almost exclusively studied in the context of patients and animal models of GA1. We showed that glutarate is present in CD8^+^ T cells and that its levels are influenced by both activation and oxygen status. We demonstrated that glutarate has an unexpected role in the regulation of cell physiology, particularly in the differentiation and metabolism of T cells. By acting as an inhibitor of αKGDDs, glutarate alters the transcriptional and epigenetic landscape of cells via inhibition of HIF-P4H and KDMs/TETs, respectively. We used CD8^+^ T cells as a model system, but note that glutarate has this inhibitory property both in cell-free assays and in a range of other cell types; thus, these effects are probably not exclusive to CD8^+^ T cells.

By altering glutarate levels in CD8^+^ T cells, either by supplementation with esterified glutarate, or by genetic manipulation of GCDH, we showed that in vitro glutarate promotes T_CM_ cell development and reduces expression of T cell exhaustion markers, while increasing cytotoxic function. Although in vivo treatment of tumour-bearing mice with DEG reduces tumour growth, while increasing the quantity of both circulating and tumour-infiltrating CD8^+^ T cells, the CD8^+^ phenotypic changes observed with ex vivo treatment with DEG were not observed in tumour-infiltrating CD8^+^ T cells. In an immune cell context, loss of TET2 expression promotes differentiation of CD8^+^ T cells into T_CM_ cells^52^ and a disrupted *TET2* gene can promote the therapeutic efficacy of CAR T cells^53^. We have illustrated that glutarate treatment of CD8^+^ T cells results in direct TET2 inhibition, correlating with both the increased levels of T_CM_ populations we see in activated CD8^+^ T cells and an increased killing of target cells.

CD8^+^ T cell differentiation from a naive quiescent cell to an effector or memory cell requires dynamic epigenetic remodelling, resulting in altered gene accessibility profiles^54^. H3K9me3 and H3K27me3 are known repressive histone modifications and are both increased in activated CD8^+^ T cells cultured with DEG, after direct competitive inhibition of KDM4C and KDM6A, respectively. H3K27me3 expression is reduced after T cell activation^5^ and in vivo studies have shown that its levels are highest in T_CM_ and naive CD8^+^ T cells^11^. In this study, we illustrated that, similarly to the effects seen after exposure to S-2HG^11^, DEG facilitates retention of H3K27me3, a marker correlated with T_CM_ cell development. Glutarate additionally inhibits the HIF prolyl hydroxylase HIF-P4H-1, albeit with a much higher IC_50_ than observed for the KDM and TET2 enzymes. Our findings indicate that glutarate is a competitive inhibitor of αKGDDs and this should be considered as a likely aspect of GA1 pathology, given the high levels of intracellular glutarate seen in that disease.

In this study, we showed that the E2 subunit of PDHc is a major target of glutarylation in CD8^+^ T cells. Glutarate in its CoA form, that is, glutaryl-CoA, acts as a substrate for glutarylation^36^. Glutarylation has important roles in mitochondrial dynamics, antioxidant defence, sperm motility, lysine oxidation and apoptotic signalling^39,55–58^. One recent study found that GCDH knockdown in melanoma resulted in programmed cell death via glutarylation of NRF2 (ref. ^55^). We did not find any evidence for increased cell death after GCDH silencing or increased glutarylation via DEG treatment in CD8^+^ T cells or in any of the other cell types that we examined in this study.

In this study, we provide evidence that lysine glutarylation of the E2 subunit of PDHc disrupts normal lipoylation, resulting in altered PDHc activity, which correlates with increased pyruvate conversion to lactate and increased glycolysis. This reaction occurs rapidly in cells treated with diethyl glutarate. Although inhibition of HIF-P4H-1 induces the transcription of genes associated with glycolysis, the levels of glutarate used in this study did not result in increased protein expression of PDHK1, in an increased PDHE1 phosphorylation or in an increased glucose uptake; this indicates that the increase in glycolytic capacity and ECAR observed in this study are probably, for the most part, independent of HIF-P4H-1 inhibition. This indicates that glutarylation represents a mechanism for PDHc control, acting via disruption of lipoylation, and demonstrates the importance of lipoate modifications in controlling the activity of PDHc^59–61^.

Constant regeneration of acetyl-CoA is essential for cells to maintain oxidative phosphorylation. Although glutarate reduces PDHc activity via glutarylation, cells treated with glutarate do not have a reduced acetyl-CoA pool, probably owing to compensation from glutarate breakdown and fatty acid oxidation. This results in maintenance of mitochondrial oxidative phosphorylation despite the reduced PDHc activity caused by glutarylation of PDHE2. The breakdown of glutarate to crotonyl-CoA, and subsequently to acetyl-CoA, generates FADH_2_, as opposed to acetyl-CoA formation from pyruvate, which generates NADH. NADH is a stronger reducing agent and enters the electron transport chain (ETC) at complex I, in contrast to FADH_2_, which enters the ETC at complex II. This differential contribution to mitochondrial membrane potential results in a reduced maximal OCR on addition of FCCP to glutarate-treated cells, indicating that, while glutarate can be used as an alternative fuel source for mitochondrial oxidation, it is not as efficient as glucose itself.

In this study, we revealed a potentially central role for glutarate as a modulator and regulator of CD8^+^ T cell metabolism and cytotoxicity. We illustrated that glutarate can influence CD8^+^ T cell differentiation and increase effector function. We showed that glutarate is an inhibitor of αKGDDs, which has many implications for better understanding the epigenetic and transcriptional landscape of cells in a catabolic state. We additionally showed that PDHE2 is a direct target of glutarate-mediated glutarylation and is involved in the control of this crucial metabolic complex. Finally, we highlighted the potential use of glutarate administration in an immunotherapeutic model, illustrating the translational potential of these findings.

## Methods

All animal experiments were performed in accordance with the ethical regulation of the UK Home Office and the University of Cambridge or the regional animal ethics committee of northern Stockholm, Sweden.

Human peripheral blood mononuclear cells (PBMCs) were obtained from the National Health Service (NHS) Blood and Transplant (NHSBT) (Addenbrooke’s Hospital, Cambridge, UK) or Karolinska Hospital Service, Sweden. Ethical approval was obtained from the East of England-Cambridge Central Research Ethics Committee (06/Q0108/281) and consent was obtained from all participants.

### Mice

C57BL/6J mice (strain 632, Charles River Laboratories), were used in the in vitro assays and in the orthotopic tumour growth and infiltration experiments (male and female mice were 8–12 weeks old). NOD.Cg-*Prkdc*^scid^*Il2rg*^tm1Wjl^/SzJ (NSG) mice (strain 005557, The Jackson Laboratory) were used in human CAR T cell experiments. Donor T cell receptor (TCR)-transgenic OT1 mice (003831, The Jackson Laboratory) were crossed with mice bearing the CD45.1 congenic marker (strain 002014, The Jackson Laboratory). Targeted deletion of HIF-1 in T cells was achieved by crossing homozygous mice carrying *loxP*-flanked *Hif1* alleles^62^ into a mouse strain of Cre recombinase expression driven by the distal promoter of the lymphocyte-specific *Lck* gene (strain 012837, The Jackson Laboratory). All experiments were performed with age-matched and sex-matched Cre-negative controls. TCR-transgenic OT1 mice and mice with targeted deletion of *HIF1* in T cells were bred and housed in specific pathogen-free conditions in accordance with the regional animal ethics committee of northern Stockholm, Sweden. All animal experiments were performed in accordance with the ethical regulation of the UK Home Office and the University of Cambridge or the regional animal ethics committee of northern Stockholm, Sweden.

### Cell lines

B16F10 cells were originally purchased from ATCC (catalogue no. CRL-6475) and genetically modified to express OVA, enhanced green fluorescent protein (GFP) and neomycin phosphotransferase^32^. The resulting OVA expression B16F10-OVA cells were cultured in DMEM (catalogue no. 11995065, Thermo Fisher Scientific) containing 0.75 mg ml^−1^ G418 sulphate (catalogue no. 10131027, Thermo Fisher Scientific). HEK 293T cells were purchased from Takara Bio (catalogue no. 632180) and cultured in DMEM. SKOV3 was purchased from ATCC (catalogue no. HTB-77) and cultured in McCoy’s 5A (Modified) Medium (catalogue no. 16600082, Thermo Fisher Scientific). Raji-GFP-Luc cells were purchased from Biocytogen (catalogue no. B-HCL-010) and cultured in Roswell Park Memorial Institute (RPMI) 1640 (catalogue no. 21875, Thermo Fisher Scientific). Jurkat cells were purchased from ATCC (catalogue no. TIB-152) and cultured in RPMI 1640. RAW 264.7 cells were purchased from ATCC (catalogue no. TIB-71) and cultured in RPMI 1640. HeLa cells were a gift from P. Lehner (University of Cambridge) and were authenticated using short tandem repeat profiling (Eurofins Genomics). HeLa cells were cultured in DMEM. MEFs were isolated from macerated embryonic day (E) 12.5–13.5 embryos. A whole litter of embryos without sex selection were used. Stable transfection with SV40 large T antigen passage 3 was used for immortalization. Cells were subcultured over 17 more passages and maintained in DMEM^63^. All media was supplemented with 10% FCS (catalogue no. 10270106, Gibco), 100 U ml^−1^ penicillin and 100 µg ml^−1^ streptomycin (catalogue no. P4333, Sigma-Aldrich). All cells were cultured in incubators with 5% CO_2_ and at either 21% or 1% oxygen, as indicated. Low oxygen incubations were performed in a Ruskinn SCI-tive workstation. Cell number and viability was determined using the ADAM-MC Automated Cell Counter (NanoEntek), CellDrop Automated Cell Counter (DeNovix) or TC20 Automated Cell Counter (Bio-Rad Laboratories).

### T cell isolation and activation

Human PBMCs were obtained from NHSBT: Addenbrooke’s Hospital, Cambridge, UK or the Karolinska Hospital Service, Sweden. Ethical approval was obtained from the East of England-Cambridge Central Research Ethics Committee (06/Q0108/281) and consent was obtained from all participants. PBMCs from healthy donors were isolated from blood using discontinuous plasma Percoll gradients. Naive CD8^+^ T cells were isolated using the Naive CD8^+^ T Cell Isolation Kit (catalogue no. 130-093-244, Miltenyi Biotec), in accordance with the manufacturer’s instructions. Total CD8^+^ T cells were purified by either positive or negative magnetic bead sorting (catalogue nos. 130-045-201 and 130-096-495, Miltenyi Biotec). All human CD8^+^ T cells were activated with anti-human CD3/CD28 Dynabeads (catalogue no. 11131D, Thermo Fisher Scientific) at a 1:1 cell:bead ratio and cultured in complete RPMI 1640 supplemented with 10% FCS, 100 U ml^−1^ penicillin, 100 µg ml^−1^ streptomycin and 30 U ml^−1^ interleukin-2 (IL-2) (catalogue no. 11147528001, Sigma-Aldrich).

Mouse CD8^+^ T cells were isolated from mouse spleens using positive selection. Incubation with microbeads conjugated to monoclonal anti-mouse CD8α (Ly-2; isotype: rat IgG2a) antibody (catalogue no. 130-049-401, Miltenyi Biotec) was followed by magnetic bead isolation on a MACS column. WT mouse CD8^+^ T cells were activated with anti-mouse CD3/CD28 Dynabeads (catalogue no. 11453D, Thermo Fisher Scientific) at a 1:1 cell:bead ratio. Purified OT1 CD8^+^ T cells were activated with 1 µg ml^−1^ of the OVA-derived peptide SIINFEKL (ProImmune). All mouse CD8^+^ T cells were cultured in complete RPMI 1640 supplemented with 10% FCS, 100 U ml^−1^ penicillin, 100 µg ml^−1^ streptomycin, 55 µM 2-ME (catalogue no. 21985023, Gibco) and 10 U ml^−1^ IL-2.

### Flow cytometry and sorting

Single-cell suspensions were stained with LIVE/DEAD Fixable Near-IR Dead Cell Stain Kit (catalogue no. 10119, Thermo Fisher Scientific) followed by surface and intracellular staining with fluorochrome-labelled antibodies (Supplementary Tables 1 and 2). Staining of cytoplasmic and nuclear antigens was performed using the Cytofix/Cytoperm Fixation/Permeabilization Kit (catalogue no. 554714, BD Biosciences) and the Transcription Factor Buffer Set (catalogue no. 562725, BD Biosciences), respectively. For the proliferation assays, cells were loaded with CellTrace Violet (catalogue no. C34557, Thermo Fisher Scientific) according to the manufacturer’s instructions. Samples were acquired on an Aurora (Cytek Biosciences).

For 5-hydroxymethylcytosine (5hmC) staining, cells were stained for surface antigens as above and then fixed and permeabilized with the Transcription Factor Buffer Set. Next cells were incubated with 4 M HCl for 10 min at room temperature. Cells were then thoroughly washed and incubated in blocking buffer (0.1% PBS-Triton X-100, 5% FCS) for 30 min at 4 °C. Cells were then incubated with primary anti-5hmC (catalogue no. 10013602, Active Motif) overnight at 4 °C and the day after with secondary antibody for 1 h at room temperature. Flow cytometry was then performed as explained above. Cells were sorted on an FACSAria III (BD Biosciences) after surface antigen staining as described above. Data were analysed using FlowJo v.10.7.2.

### ELISA

Secreted IFNγ levels of cultured cells were determined using an ELISA. Assays were performed with uncoated ELISA kits (catalogue no. 88-7316, Thermo Fisher Scientific), according to the manufacturer’s instructions. Absorbance was determined using a microplate reader (Sunrise, Tecan) at a wavelength of 450 nm.

### Lentivirus transduction

To generate lentiviral particles, 5 × 10^6^ HEK 293 cells were plated in 15-cm Petri dishes and transfected the day after with 50 µl FuGENE (catalogue no. E2311, Promega Corporation), 10 µg CAR-encoding vectors and 3.3 µg of each third-generation lentivirus helper vectors (catalogue no. CART-027CL, Creative-Biolabs). Supernatant containing lentiviral particles was collected 48 h after transfection and used fresh or stored at −80 °C. Lentiviral supernatant was spun onto non-treated plate wells coated with 30 µg ml^−1^ RetroNectin reused up to three times (catalogue no. T100B, Takara Bio) at 2,000*g* for 2 h at 32 °C and replaced with activated human CD8^+^ T cells in fresh RPMI 1640 supplemented with 30 U ml^−1^ IL-2.

### In vitro cytotoxicity

Ten thousand B16F10-OVA or SKOV3 cells were seeded per well in 96-well plates and cocultured for a minimum of 14 h with varying ratios of mouse CD8^+^ OT1 or human CD8^+^RQR8^+^ HER2 CAR T cells, respectively. T cells had been treated with or without DEG 7 days before the assay. Wells were washed twice with PBS to remove T cells and the number of remaining target cells was determined by culturing with 10 µg ml^−1^ resazurin (catalogue no. R7017, Sigma-Aldrich) and measuring the fluorescence signal in a plate reader. Cytotoxicity was calculated relative to wells with no T cells added. Ten thousand Raji cells were cocultured for a minimum of 14 h with CD19 CAR T cells previously activated for 7 days and cultured with or without DEG. Cytotoxicity was assessed by flow cytometry using the ratio of live Raji cells to CountBright Absolute Counting Beads (catalogue no. C36950, Thermo Fisher Scientific). To determine specific cytotoxicity, data were normalized to the cytotoxicity of the vector-controlled transduced CD8 T cells of the respective donor.

### Vectors

DNA encoding a codon-optimized polycistronic peptide consisting of RQR8 and anti-human HER2 (clone 4D5) interspersed with picornavirus T2A and furin cleavage sequences was synthesized by GeneScript. RQR8, used as the transduction marker, is a chimeric surface protein consisting of domains from CD34 (for detection and purification with clone QBEND/10), CD8 (for anchoring at the cell surface) and CD20 (for depletion in vivo with the anti-CD20 monoclonal antibody rituximab)^64^. shRNA sequences corresponding to the best specificity score for each target were retrieved from the RNAi Consortium library. For coexpression of protein and shRNA, shRNA hairpins were flanked with an optimized sequence of miR-30 (refs. ^65,66^). 97-mer oligonucleotides (IDT Ultramers) coding for the respective shRNAs^67^ were PCR-amplified using 10 µM of the primers miRE-XhoI-fw (5′- TGAACTCGAGAAGGTATATTGCTGTTGACAGTGAGCG-3′) and miRE-EcoRI-rev (5′-TCTCGAATTCTAGCCCCTTGAAGTCCGAGGCAGTAGGC-3′), a 0.5-ng oligonucleotide template and the Q5 High-Fidelity 2X Master Mix (New England Biolabs) and cloned into HER2 CAR vectors containing the miRE scaffold sequence. All coding sequences were cloned into pCDCAR1 (Creative Biolabs). Third-generation lentiviral transfer helper plasmids were obtained from Biocytogen. All sequences are available in Supplementary Table 3.^65,67^ The following lentiviral vectors purchased from Creative Biolabs were used: a truncated form of epidermal growth factor receptor (vector control) and anti-CD19 CAR with a 4-1BB endodomain.

### Metabolite extraction and liquid chromatography–tandem mass spectrometry analysis

#### Metabolite extraction and liquid chromatography–tandem mass spectrometry analysis

Cells were counted to determine viable cell numbers (Fig. 1f,h,i and Extended Data Fig. g,h,k). A total of 0.5–2 × 10^6^ viable cells were collected, washed with cold PBS and metabolic activity was quenched by freezing samples in dry ice and ethanol, and stored at −80 °C. Metabolites were extracted by adding 150 μl ice-cold 1:1 (vol/vol) methanol/water (containing 1 mM *N*-cyclohexyl-2-aminoethanesulfonic acid (CHES) as an internal standard) to the cell pellets; samples were transferred to a chilled microcentrifuge tube containing 150 μl chloroform and 500 μl methanol (750 μl total in 3:1:1 vol/vol methanol/water/chloroform). Samples were sonicated in a water bath for 8 min at 4 °C, and centrifuged (13,000*g*) for 10 min at 4 °C. The supernatant containing the extract was transferred to a new tube for evaporation using TurboVap LV (Biotage), resuspended in 3:3:1 (vol/vol/vol) methanol/water/chloroform (350 μl total) to phase-separate polar metabolites from apolar metabolites and centrifuged. The aqueous phase was transferred to a new tube for evaporation using TurboVap LV, washed with 60 μl methanol and dried again. Evaporated extracts were dissolved in 100 µl MeOH–50% water, before injection on the machine. Isotopically labelled glutarates were determined by liquid chromatography (LC)–tandem mass spectrometry (tandem MS) on a Waters ACQUITY UPLC system coupled to a Xevo-TQ-XS mass spectrometer. A SeQuant ZIC-HILIC column (2.1 × 100 mm, 3.5 μm, Merck) equipped with a guard column (ZIC-HILIC Guard, 20 × 2.1 mm) was used for the separation, with mobile phase A consisting of MilliQ water, 0.1% v/v formic acid (FA) and mobile phase B consisting of acetonitrile, 0.1% v/v FA The column was thermostated at 40 °C, the injection volume was set to 3 µl and the gradient was carried out at a flow rate of 0.3 ml min^−1^ as follows: 5% A from 0 to 1 min, linearly increased to 30% from minutes 1–5 and to 75% at minute 5.5. The column was subsequently washed in 75% A for 2.5 min and re-equilibrated at initial conditions for 3 min. MS analyses were performed in multiple reaction monitoring (MRM) mode, with positive electrospray ionization (ESI) from minute 0 – 1.4 and negative electrospray ionization (ESI) from minute 1.4 to minute 4.7. Positive ESI was conducted according to the following parameters: capillary potential 3 kV, source offset 30 V, cone voltage 15 V, source temperature 150 °C, desolvation temperature 600 °C, cone gas flow 150 l h^−1^, desolvation gas flow, 1,000 l h^−1^, nebulizer 7 bar and collision gas flow 0.17 ml min^−1^. Negative ESI was operated under the same conditions but with a capillary potential set to 2 kV and a cone voltage set to 20 V. Acquisition was performed using MRM with the mass transitions and parameters illustrated in Supplementary Table 4. Two transitions were used for each of the target compounds and the ion ratio between them was used to increase the confidence in the identification. To account for instrumental variability, 1 mM CHES was spiked in the samples before cell lysation to be used as the volumetric internal standard.

#### Metabolite extraction and LC–MS analysis

Cells were counted to determine the viable cell numbers (Extended Data Fig. 1i,n). A total of 0.5–2 × 10^6^ viable cells were collected, washed with cold PBS and metabolic activity was quenched by freezing samples in dry ice and ethanol and stored at −80 °C. Before extraction, all samples were added with 10 µl of the internal standard solution, consisting of 200 µg ml^−1^ succinic acid-d4. Metabolites were subsequently extracted by adding 1,000 μl ice-cold LC–MS-grade methanol to the cell pellets, followed by 30 min of sonication. Sonication was carried out by adding ice to the ultrasound bath to maintain the temperature below 20 °C. After sonication, samples were centrifuged (12,000*g*, 20 min, 6 °C) and 200 µl of the supernatant were transferred to LC–MS vials for the subsequent analysis.

Glutarate was determined by LC–tandem MS on a Waters ACQUITY UPLC system coupled to a Xevo-TQ-S mass spectrometer (Waters). An ACQUITY Premier BEH Amide Vanguard Fit column (2.1 × 100 mm, 1.7 μm), equipped with an integrated guard column, was used for the separation, with mobile phase A consisting of 20 mM ammonium formate + 0.1% FA in double deionized water and mobile phase B consisting of 0.1% FA in acetonitrile. The column was thermostated at 30 °C, the injection volume was set to 2 µl and the gradient was carried out at a flow rate of 0.35 ml min^−1^ as follows: 95% B from 0 to 1 min, linearly decreased to 55% B from minute 1 to minute 5 and to 45% B from minute 5 to minute 7, and increased back to 95% B in minutes 7–8.5. The column was subsequently re-equilibrated at initial conditions until minute 15.

MS analyses were performed using ESI with the following parameters: capillary potential −1.5 kV, source temperature 150 °C, desolvation temperature 550 °C, cone gas flow 150 l h^−1^, desolvation gas flow 800 l h^−1^, nebulizer 7 bar and collision gas flow 0.17 ml min^−1^. Two selected reaction monitoring transitions were acquired for glutarate (131.0 > 69.2 and 131.0 > 113.1 as quantifier and qualifier transitions, respectively). The ion ratio between the transitions was used as an additional confirmation of the identification. For succinate-d_4_, the 121.0 > 58.1 transition was used.

### qPCR

Total RNA was extracted from isolated CD8^+^ T cells (RNeasy Kit, QIAGEN); 300 ng of RNA were used for complementary DNA synthesis (First-Strand cDNA Synthesis Kit, Invitrogen). Quantitative PCR with reverse transcription (RT–qPCR) was performed with SYBR green (Roche) in a StepOnePlus system (Applied Biosystems). All kits were used according to the manufacturer’s instructions. Samples were run in technical duplicates. Primers were designed with NCBI primer blast and are listed in Supplementary Table 5.

### Western blot

Cell pellets were lysed by either urea-Tris buffer (8 M urea, 50 mM Tris-HCl (pH 7.5), 150 mM β-mercaptoethanol), sonicated twice for 45 s intercalated with 1 min incubation on ice and centrifuged at 14,000*g*, 4 °C for 15 min or by RIPA Lysis and Extraction Buffer (catalogue no. 89900, Thermo Fisher Scientific), according to the manufacturer’s instructions. Nuclear and cytosolic fractions were prepared from cells using the NE-PER kit (catalogue no. 78833, Thermo Fisher Scientific). Histones were extracted with the Histone Extraction Kit (catalogue no. ab113476, Abcam). Proteins were separated by SDS–PAGE and transferred to polyvinylidene fluoride (PVDF) membranes. Total protein stain was obtained using non-stain protein labelling reagent (catalogue no. A44448, Thermo Fisher Scientific). Membranes were then blocked in 5% milk prepared in PBS plus 0.05% Tween-20, incubated with primary antibodies overnight at 4 °C and horseradish peroxidase (HRP)-conjugated secondary antibodies (catalogue nos. HAF008 and HAF007, R&D Systems) for 1 h the next day. After enhanced chemiluminescence (ECL) exposure (catalogue no. GERPN2106, Sigma-Aldrich), membranes were imaged using an iBrightCL1000 (Thermo Fisher Scientific). The following primary antibodies from Cell Signaling Technology were used at a concentration of 1:1,000: anti-PDHK1 (catalogue no. 3820); anti-β-actin (catalogue no. 12262S); anti-PPIB (catalogue no. 43603); anti-OGDH (catalogue no. 26865S); anti-phospho-PDH α1 (catalogue no. 37115); anti-SIRT5 (catalogue no. 8779S); anti-H3 (catalogue no. 4499); anti-H3K4me2 (catalogue no. 9725); anti-H3K4me3 (catalogue no. 9751); anti-H3K9me2 (catalogue no. 4658); anti-H3K9me3 (catalogue no. 13969); anti-H3K27me2 (catalogue no. 9728); anti-H3K27me3 (catalogue no. 9733); anti-H3K36me2 (catalogue no. 2901); anti-H3K36me3 (catalogue no. 4909); anti-H3K79me2 (catalogue no. 5427); anti-H3K79me3 (catalogue no. 4360); anti-H3k9ac (catalogue no. 9649); anti-H3K18ac (catalogue no. 13998); and anti-H3k27ac (catalogue no. 8173). The following antibodies from Thermo Fisher Scientific were used at a concentration of 1:1,000: PDHc (catalogue no. 456799) and GCDH (catalogue no. PA5-60294). Other antibodies used at a concentration of 1:1,000 are as follows: pan anti-glutarylysine (catalogue no. PTM-1151, PTM Biolabs) and anti-lipoic acid (catalogue no. 437695, Sigma-Aldrich).

For phosphorylation western blot analysis, stripping buffer (catalogue no. 21056, Thermo Fisher Scientific) was used for 15 min before membranes were re-blocked and stained for total protein as above.

Lipoic acid western blotting was performed as above with the following alterations: blocking buffer and secondary antibody buffer (5% milk plus 1% fatty acid free bovine serum albumin (BSA) (catalogue no. BP9704100, Thermo Fisher Scientific) prepared in PBS plus 0.05% Tween-20); and primary antibody buffer (2% fatty acid free BSA prepared in PBS plus 0.05% Tween-20). Lipoic acid antibody was used at a concentration of 1:2,000 (catalogue no. 437695, Sigma-Aldrich). Data were analysed using the iBright Analysis Software v.4.0.1 or ImageJ.

### In vitro TET2 and KDM4C enzymatic activity assay

A total of 2 nM of the human TET2 enzyme (catalogue no. 50162, BPS Bioscience) or 4 nM of the human KDM4C enzyme (catalogue no. 50105, BPS Bioscience) was incubated with the substrate of 30 nM single-stranded DNA (ssBiotin 26 nt Me-C Oligo, Genscript), in the presence of αKG (catalogue no. K3752, Sigma-Aldrich), 10 µM ammonium iron(II) sulphate hexahydrate (catalogue no. F3754, Sigma-Aldrich) in assay buffer (50 mM HEPES (catalogue no. 15630-080, Gibco), pH 7.0, 100 mM NaCl, 0.01% Pluronic F-127, 1 mM TCEP (S16054, Shanghai Yuanye Bio-Technology Company), 2 mM ascorbic acid (A5960, Sigma-Aldrich), 0.2 mg/ml BSA (catalogue no. B2064, Sigma-Aldrich) and 1,000 U ml^−1^ catalase (catalogue no. C40, Sigma-Aldrich)). The concentration of αKG used for the TET2 standard and competitive assays was 115 µM and 4,500 µM, respectively. The concentration of αKG used for the KDM4C standard and competitive assays was 10 µM and 50 µM, respectively. The concentration of αKG used in the standard assay was calculated for each enzyme batch and is the concentration of substrate at which half the maximum reaction rate is achieved. DEG and glutaric acid were used in threefold serial dilution and the maximum concentration used was 5 mM. The pre-incubation time of the inhibitors with the TET2 mixture was 30 min at room temperature. The reaction step was at room temperature for 90 min. The product was detected by using a 5 nM anti-5hmC antibody (catalogue no. 39999, Active Motif), 5 nM Eu-Protein A (catalogue no. 61PRAKLB, Cisbio), 6.25 nM streptavidin-Alexa Fluor 647 (catalogue no. S21374, Thermo Fisher Scientific) and 10 mM EDTA (catalogue no. 0369, Sigma-Aldrich). For the standard curve, the ssBiotin 26 nt HydMe-C Oligo was used. The assays were performed in technical duplicates in a 384-well plate. Inhibitor analysis was performed within the linear range of catalysis.

The percentage of inhibition was calculated with the following formula: inhibition% = (1−(signal value per well−average low control)/(average high control−average low control)) × 100. Data were fitted using Prism (GraphPad Software) with a four-parameter equation via ‘log(inhibitor) versus response−variable slope’ model.

### In vitro HIF-P4H-1 and KDM6A activity assays

The human full-length HIF-P4H-1 and KDM6A enzymes were recombinantly produced using Sf21 insect cells and the enzymes were purified with anti-FLAG M2 affinity gel as described previously^68,69^. Briefly, the C terminus FLAG-tagged human HIF-P4H1-2 and KDM6A were generated using PCR and subcloned into pAcG3X and pVL1393 baculovirus expression vectors, respectively. The recombinant baculoviruses were generated by transducing the bacmid DNA into *Spodoptera frugiperda* Sf9 cells using BaculoGold DNA (HIF-P4H1-2, BD Biosciences) and BacMagic-3 DNA kit (for KDM6A, Novagen). Recombinant proteins were produced by infecting Sf21 insect cells with the corresponding amplified baculoviruses for 72 h at 27 °C. Cells were homogenized in a buffer containing 10 mM Tris, 150 mM NaCl, 100 mM glycine, 0.1% (v/v) Triton X-100, pH 7.8, and a protease inhibitor cocktail tablet without EDTA. The cell lysates were centrifuged at 21,000*g* for 30 min; the soluble fractions containing the FLAG-tagged proteins were affinity-purified using the anti-FLAG M2 affinity gel (Sigma-Aldrich). The gel beads were washed with TBS buffer (50 mM Tris, 150 mM NaCl, pH 7.4, protease inhibitor cocktail tablet without EDTA) and the proteins were eluted with TBS buffer containing additionally 150 µg ml^−1^ FLAG peptide. Protein concentration was measured with NanoDrop and proteins were analysed by SDS–PAGE followed by Coomassie brilliant blue staining. The inhibition assays were carried out using a method that measures the hydroxylation-coupled stoichiometric release of ^14^CO_2_ from 2-oxo-[1-^14^C] glutarate with a synthetic HIF-1α peptide or a histone H3K27me3 peptide as substrates for HIF-P4H-1 and HIF-P4H-2 and KDM6A, respectively^68,69^. Glutarate was used at increasing concentrations (50 µM to 10 mM) in the assay. The concentration of 2-oxoglutarate was 8 µM and 32 µM for the HIF-P4H-1 and KDM6A catalysed reactions, respectively, while keeping the concentrations of other cosubstrates and cofactors saturating and constant. The IC_50_ value of the glutarate analogue was calculated from the inhibition saturation curves.

### HIF-PH cellular activity assays

MEFs were transiently transfected using Lipofectamine 2000 (Thermo Fisher Scientific) with 20 ng per expression plasmid for 2 × 10^4^ cells per well in a 96-well plate. Cells were treated with transfection mixes for 8 h.

Generation of plasmids for the HIF-PH activity luciferase reporter assay has been described previously: luciferase reporter driven by GRE-luc pFLAG-Gal4-mHIF-1α NTAD^35^. Briefly, cells were concurrently transfected with pGRE-luc and a plasmid encoding constitutive expression of a fusion protein linking a FLAG-tagged Gal4 DNA-binding domain with one of the transcription activation domains of murine HIF-1α. The NTAD contains proline residues that are hydroxylated by HIF-PH enzymes to target the protein for degradation; thus, luciferase expression is controlled by HIF-PH hydroxylation activity against the pFLAG-Gal4-mHIF-1α NTAD. After 8 h of transient transfection with expression plasmids, cells were treated with experimental medium containing metabolites and drugs as described in the figure legends for 16 h; then, the luciferase signal assessed using the Steady-Glo Luciferase Assay System (Promega Corporation) detected using a luminometer. The luciferase signal was normalized to that induced by 2.5 mM DMOG.

### IP of CD8^+^ T cells

A total of 30 × 10^6^ CD8^+^ T cells were lysed with Pierce IP Lysis Buffer (catalogue no. 87787, Thermo Fisher Scientific). IP was performed using protein A Dynabeads (catalogue no. 10002D, Thermo Fisher Scientific) according to the manufacturer’s instructions; 1–10 µg of target antibody was used per reaction. Eluted proteins were separated using SDS–PAGE, the gel was stained with InstantBlue Coomassie Protein Stain (catalogue no. ab119211, Abcam), imaged using an iBrightCL1000 and the stained target bands were cut and stored in water until analysis. Non-cut gels were transferred to PVDF membranes and then blocked in 5% milk prepared in PBS plus 0.05% Tween-20. Membranes were then incubated with primary antibodies overnight at 4 °C and HRP-conjugated secondary antibodies (HAF008 and HAF007) for 1 h the next day. After ECL exposure, membranes were imaged using an iBrightCL1000. The following primary antibodies were used at a concentration of 1:1,000; PDHc, OGDH and pan anti-glutarylysine.

### In-gel protein digestion and MS

Protein bands were excised manually from gels and in-gel digested using a MassPREP robotic protein-handling system (Waters). Gel pieces were distained according to the manufacturer’s description. Then, proteins were reduced with 10 mM dithiothreitol (DTT) in 100 mM Ambic for 30 min at 40 °C and alkylated with 55 mM iodoacetamide in 100 mM Ambic for 20 min at 4 °C followed by digestion with 0.3 mg trypsin (sequence grade, Promega Corporation) in 50 mM Ambic for 5 h at 4 °C. The tryptic peptides were extracted with 1% FA in 2% acetonitrile, followed by 50% acetonitrile twice. The liquid was evaporated to dryness on a vacuum concentrator (Eppendorf).

The reconstituted peptides in solvent A (2% acetonitrile, 0.1% FA) were separated on a 50-cm long EASY-spray column (Thermo Fisher Scientific) connected to an UltiMate 3000 Nano-LC system (Thermo Fisher Scientific) using a 60-min gradient from 4% to 26% of solvent B (98% acetonitrile, 0.1% FA) in 55 min and up to 95% of solvent B in 5 min at a flow rate of 300 nl min^−1^. Mass spectra were acquired on a Q Exactive HF Hybrid Orbitrap mass spectrometer (Thermo Fisher Scientific) in *m/z* 375–1,500 at a resolution of *R* = 120,000 (at *m/z* 200) for full mass, followed by data-dependent higher-energy C-trap dissociation (HCD) fragmentations from 17 most intense precursor ions with a charge state 2+ to 7+. The tandem mass spectra were acquired with a resolution of *R* = 30,000, targeting 2 × 10^5^ ions, setting the isolation width to *m/z* 1.4 and normalized collision energy to 28%.

Acquired raw data files were analysed using the Mascot Server v.2.5.1 (Matrix Science) and searched against the SwissProt protein database (20,368 human entries). A maximum of two missed cleavage sites were allowed for trypsin, while setting the precursor and the fragment ion mass tolerance to 10 ppm and 0.02 Da, respectively. Dynamic modifications of oxidation on methionine, deamidation of asparagine and glutamine, and acetylation of the *N* termini were set. Initial search results were filtered with a 5% false discovery rate using Percolator to recalculate Mascot scores. Protein identifications were accepted if they could be established at greater than 96.0% probability and contained at least two identified peptides. Proteins that contained similar peptides and could not be differentiated based on tandem MS analysis alone were grouped to satisfy the principles of parsimony.

### IP and MS detection of lipoylation and glutarylation

HeLa cells were lysed on ice for 30 min in a HEPES buffer (150 mM NaCl, 50 mM HEPES, pH 7.0, 1% IGEPAL CA-630, 1 mM nicotinamide (Sigma-Aldrich), 5 mM Tris(2-carboxyethyl)phosphine hydrochloride (Sigma-Aldrich), 1× Complete Protease Inhibitor Cocktail (Roche) and 1 mM phenylmethylsulfonyl fluoride). Lysates were centrifuged at 14,000*g* for 10 min at 4 °C. Free thiols were blocked by adding 10 mM *N*-ethylmaleimide (NEM) (Sigma-Aldrich) and incubated on a rotator at 4 °C for 1 h. Lysates were immunoprecipitated by incubation with protein G Dynabeads (catalogue no. 10003D, Invitrogen), first for 1 h to pre-clear, and then overnight with the DLAT antibody (12362S (4A4-B6-C10), Cell Signaling Technology). Resins were washed four times with Tris-buffered saline containing 0.1% IGEPAL CA-630, followed by three washes with Tris-buffered saline. Protein was eluted using 2% SDS, 50 mM Tris, pH 7.4, 150 mM NaCl, 1 mM DTT and 10% glycerol, and incubated at 90 °C for 5 min. Proteins were then separated using NuPAGE 4–12% Bis-Tris gel (catalogue no. NP0321BOX, Invitrogen), and visualized using SimplyBlue SafeStain (Invitrogen).

Mass spectrometry data were acquired using an Orbitrap Fusion Lumos coupled to an UltiMate 3000 RSLCnano UHPLC equipped with a 100-µm ID × 2 cm Acclaim PepMap Precolumn (Thermo Fisher Scientific) and a 75-µm ID × 50 cm, 2-µm particle Acclaim PepMap RSLC analytical column. Loading solvent was 0.1% FA with analytical solvents A: 0.1% FA and B: 80% MeCN + 0.1% FA. Samples were loaded at 5 µl min^−1^ loading solvent for 5 min before beginning the analytical gradient. The analytical gradient was 3–40% B over 42 min rising to 95% B by 45 min followed by a 4-min wash at 95% B and equilibration at 3% solvent B for 10 min. Columns were held at 40 °C. Data were acquired in a DDA fashion with the following settings: MS1: 380–1,500 Th, 120,000 resolution, 4 × 10^5^ AGC target, 50-ms maximum injection time; MS2: quadrupole isolation at an isolation width of *m/z* 1.6, HCD fragmentation (NCE 30) with fragment ions scanning in the Orbitrap from *m/z* 110, 5 × 10^4^ AGC target, 100-ms maximum injection time. Dynamic exclusion was set to ±10 ppm for 60 s. MS2 fragmentation was trigged on precursors 5 × 10^4^ counts and above.

Raw files were processed using PEAKS Studio (v.8.0, Bioinformatics Solutions) with the following parameters: enzyme: AspN, human database (UniProt reference proteome downloaded 21 January 2021 containing 20,541 proteins) with additional contaminant database (containing 246 common contaminants); variable modifications at PEAKS database stage: oxidation, carbamidomethylation, lipoylation, 1× and 2× NEM, glutaryl lipoate, NEM glutaryl lipoate and 309 built-in modifications at PEAKS PTM stage. The area under the curve (AUC) values for each peptide were extracted from the PEAKS peptide list, with AUCs calculated by PEAKS.

### PDHc activity assay

PDHc activity was determined using a PDHc Activity Assay Kit (catalogue no. MAK183, Sigma-Aldrich). A total of 1 × 10^6^ cells were used per reaction and the assay was performed according to the manufacturer’s instructions. Activity was normalized to total protein concentration. Data were acquired on a FLUOstar Omega (BMG Labtech).

### Seahorse

A Seahorse XFe bioanalyzer was used to measure the OCR and ECAR. A total of 1.5 × 10^5^ CD8^+^ T cells per well were spun onto poly-D-lysine-coated (catalogue no. P7280, Sigma-Aldrich) Seahorse plates and preincubated at 37 °C for a minimum of 30 min in the absence of CO_2_ in Seahorse XF RPMI medium, pH 7.4 (10376-100, Agilent), supplemented with 10 mM glucose (catalogue no. A2494001, Thermo Fisher Scientific) and 2 mM glutamine (catalogue no. 25030081, Thermo Fisher Scientific). A minimum of five technical replicates per biological replicate were used. For the MST and GST, OCR and ECAR were measured under basal conditions and after the addition of the following: 750 µM DEG (catalogue no. QB-1473, Combi-block), 0.05% DMSO, 1 μM oligomycin (catalogue no. 75351, Sigma-Aldrich), 1.5 μM FCCP (catalogue no. C2920, Sigma-Aldrich), 100 nM rotenone + 1 μM antimycin A (catalogue nos. R8875 and A8674, Sigma-Aldrich), 10 mM glucose and 50 mM 2-DG (catalogue no. B1048-100, BioVision) as indicated. To assess mitochondrial fuel use, OCR was measured subsequent to the addition of the following drugs in different combinations as indicated: 3 µM BPTES (catalogue no. SML0601, Sigma-Aldrich), 2 µM UK-5099 (catalogue no. PZ0160, Sigma-Aldrich) and 4 µM etomoxir (catalogue no. E1905, Sigma-Aldrich). Assay parameters were as follows: 3 min mix, no wait, 3 min measurement, repeated 3–5 times at basal and after each addition. Measurements were taken using a 96-well Extracellular Flux Analyzer (Seahorse Bioscience).

### Glucose uptake assay

Glucose uptake was determined using a Glucose Uptake Assay Kit (catalogue no. J1341, Promega Corporation). The assay was performed according to the manufacturer’s instructions. Data were acquired on the FLUOstar Omega.

### Acetyl-CoA measurement

Acetyl-CoA levels were determined using an acetyl-CoA assay kit (catalogue no. MAK039, Sigma-Aldrich). After culture, 2 × 10^6^ cells per condition were collected, washed twice with ice-cold PBS and resuspended in 500 µl of the assay buffer. Following deproteinization by adding 2 µl 1M perchloric acid, samples were sonicated and the assay was performed according to the manufacturer’s instructions. Data were acquired on the FLUOstar Omega.

### Orthotopic tumour growth and infiltration experiments

Eight to 15-week-old female NSG mice were inoculated subcutaneously with 1 × 10^6^ SKOV3 and injected 21 days later with 5 × 10^5^ RQR8^+^ human CD8^+^ CAR T cells expanded for seven days ex vivo. 100 U of IL-2 was injected peritoneally in NSG mice on the day of ACT and 3 days later. Animals were assigned randomly to each experimental group and tumour measurements were blinded. Tumour volume (a × b × b/2, where a is the length and b is the width) was measured every two to 3 days with electronic callipers until day 80. Tumour experiments using NSG mice were carried out with approval from the regional animal ethics committee of northern Stockholm, Sweden where the permitted maximal tumour volume of 1,000 cm^3^ was not exceeded.

Seven to 10-week-old male C57BL/6J mice were inoculated subcutaneously with 1.0 × 10^6^ B16F10-OVA. Animals were assigned randomly to each experimental group. Four days after inoculation, mice were treated with a peritoneal injection of 0.2 mg DEG (approximately 10 mg kg^−1^). Treatment was repeated every 2–3 days until day 28. Tumour volume was measured every 2–3 days with electronic callipers until day 30. Peripheral blood was collected from the tail vein at day 7 and 14 days after tumour inoculation and analysed using flow cytometry. On day 14, tumours were dissected and dissociated using the Tumor Dissociation Kit (catalogue no. 130-095-929, Miltenyi Biotec), according to the manufacturer’s instructions. Spleen and the tumour-draining lymph node were mashed in cell strainers. The tumour, spleen and draining lymph node single-cell suspensions were stained with fluorochrome-labelled antibodies and analysed using flow cytometry. The tumour experiments using C57BL/6J mice were carried out in accordance with the ethical regulations of the UK Home Office and the maximal tumour burden of 1,500 cm^3^ was not exceeded (mice were euthanized when tumour volume exceeded 1,000 cm^3^).

### Statistics

Statistical analyses were performed with Prism 9 (GraphPad Software). *P* < 0.05 was considered statistically significant; the statistical tests used are stated in the figure legends.

### Reporting summary

Further information on research design is available in the Nature Portfolio Reporting Summary linked to this article.

### Supplementary information

Supplementary Information Supplementary Tables 1–5.

Reporting Summary

### Source data

Source Data Fig. 2 Unprocessed western blots.

Source Data Fig. 3 Unprocessed western blots and gels.

Source Data Extended Data Fig. 1 Unprocessed western blots.

Source Data Extended Data Fig. 2 Unprocessed western blots.

Source Data Extended Data Fig. 3 Unprocessed western blots and gels.

Source Data Extended Data Fig. 4 Unprocessed western blots.

## Data Availability

All data generated or analysed during this study are included in the published article and its supplementary information files. The data that support the findings of this study are available from the corresponding author upon reasonable request. Source data are provided with this paper.

## Code availability

No code was used in this study.

## References

[1] Kaech SM, Cui W (2012). Transcriptional control of effector and memory CD8^+^ T cell differentiation. Nat. Rev. Immunol..

[2] Sallusto F, Lanzavecchia A, Araki K, Ahmed R (2010). From vaccines to memory and back. Immunity.

[3] Crompton JG (2016). Lineage relationship of CD8^+^ T cell subsets is revealed by progressive changes in the epigenetic landscape. Cell Mol. Immunol..

[4] Harland KL (2014). Epigenetic plasticity of Cd8a locus during CD8^+^ T-cell development and effector differentiation and reprogramming. Nat. Commun..

[5] Russ BE (2014). Distinct epigenetic signatures delineate transcriptional programs during virus-specific CD8^+^ T Cell differentiation. Immunity.

[6] Wei J, Raynor J, Nguyen T-LM, Chi H (2017). Nutrient and metabolic sensing in T cell responses. Front. Immunol..

[7] Kedia-Mehta N, Finlay DK (2019). Competition for nutrients and its role in controlling immune responses. Nat. Commun..

[8] Palazon A (2017). An HIF-1α/VEGF-A axis in cytotoxic T cells regulates tumor progression. Cancer Cell.

[9] Ross SH, Rollings CM, Cantrell DA (2021). Quantitative analyses reveal how hypoxia reconfigures the proteome of primary cytotoxic T lymphocytes. Front. Immunol..

[10] Caldwell CC (2001). Differential effects of physiologically relevant hypoxic conditions on T lymphocyte development and effector functions. J. Immunol..

[11] Tyrakis PA (2016). S-2-hydroxyglutarate regulates CD8^+^ T-lymphocyte fate.. Nature.

[12] Foskolou IP (2020). The S enantiomer of 2-hydroxyglutarate increases central memory CD8 populations and improves CAR-T therapy outcome. Blood Adv..

[13] Bunse L (2018). Suppression of antitumor T cell immunity by the oncometabolite (R)-2-hydroxyglutarate. Nat. Med..

[14] Laukka T (2016). Fumarate and succinate regulate expression of hypoxia-inducible genes via TET enzymes. J. Biol. Chem..

[15] Koivunen P (2007). Inhibition of hypoxia-inducible factor (HIF) hydroxylases by citric acid cycle intermediates: possible links between cell metabolism and stabilization of HIF. J. Biol. Chem..

[16] Koivunen P (2012). Transformation by the (*R*)-enantiomer of 2-hydroxyglutarate linked to EGLN activation. Nature.

[17] Chowdhury R (2011). The oncometabolite 2-hydroxyglutarate inhibits histone lysine demethylases. EMBO Rep..

[18] Xiao M (2012). Inhibition of α-KG-dependent histone and DNA demethylases by fumarate and succinate that are accumulated in mutations of FH and SDH tumor suppressors. Gene Dev..

[19] Baksh SC, Finley LWS (2020). Metabolic coordination of cell fate by α-ketoglutarate-dependent dioxygenases. Trends Cell Biol..

[20] Losman J-A, Koivunen P, Kaelin WG (2020). 2-Oxoglutarate-dependent dioxygenases in cancer. Nat. Rev. Cancer.

[21] Morris JP (2019). Ketoglutarate links p53 to cell fate during tumour suppression. Nature.

[22] Gholson RK, Sanders DC, Henderson LM (1959). Glutaric acid: a product of tryptophan metabolism. Biochem. Biophys. Res. Commun..

[23] Borsook H, Deasy CL, Haagen-Smit AJ, Keighley G, Lowy PH (1948). The degradation of α-aminoadipic acid in guinea pig liver homogenate. J. Biol. Chem..

[24] Dwyer TM, Rao KS, Goodman SI, Frerman FE (2000). Proton abstraction reaction, steady-state Kinetics, and oxidation−reduction potential of human glutaryl-CoA dehydrogenase. Biochemistry.

[25] Marlaire S, Schaftingen EV, Veiga-da-Cunha M (2013). C7orf10 encodes succinate-hydroxymethylglutarate CoA-transferase, the enzyme that converts glutarate to glutaryl-CoA. J. Inherit. Metab. Dis..

[26] Goodman SI, Markey SP, Moe PG, Miles BS, Teng CC (1975). Glutaric aciduria; A “new” disorder of amino acid metabolism. Biochem. Med..

[27] Strauss KA, Puffenberger EG, Robinson DL, Morton DH (2003). Type I glutaric aciduria, part 1: natural history of 77 patients. Am. J. Med. Genet. C Semin. Med. Genet..

[28] Kyllerman M (2004). Long-term follow-up, neurological outcome and survival rate in 28 Nordic patients with glutaric aciduria type 1. Eur. J. Paediatr. Neurol..

[29] Kölker S, Ahlemeyer B, Krieglstein J, Hoffmann GF (2000). Evaluation of trigger factors of acute encephalopathy in glutaric aciduria type I: fever and tumour necrosis factor-alpha. J. Inherit. Metab. Dis..

[30] Howden AJM (2019). Quantitative analysis of T cell proteomes and environmental sensors during T cell differentiation. Nat. Immunol..

[31] Lukashev D, Caldwell C, Ohta A, Chen P, Sitkovsky M (2001). Differential regulation of two alternatively spliced isoforms of hypoxia-inducible factor-1α in activated T lymphocytes. J. Biol. Chem..

[32] Veliça P (2021). Modified hypoxia-inducible factor expression in CD8^+^ T cells increases antitumor efficacy. Cancer Immunol. Res..

[33] Sekine T (2020). TOX is expressed by exhausted and polyfunctional human effector memory CD8^+^ T cells. Sci. Immunol..

[34] Khan O (2019). TOX transcriptionally and epigenetically programs CD8^+^ T cell exhaustion. Nature.

[35] Pereira T, Zheng X, Ruas JL, Tanimoto K, Poellinger L (2003). Identification of residues critical for regulation of protein stability and the transactivation function of the hypoxia-inducible factor-1α by the von Hippel–Lindau tumor suppressor gene product. J. Biol. Chem..

[36] Tan M (2014). Lysine glutarylation is a protein posttranslational modification regulated by SIRT5. Cell Metab..

[37] Bao X (2019). Glutarylation of histone H4 lysine 91 regulates chromatin dynamics. Mol. Cell.

[38] Xie L (2021). Functions and mechanisms of lysine glutarylation in eukaryotes. Front. Cell Dev. Biol..

[39] Schmiesing J (2018). Disease-linked glutarylation impairs function and interactions of mitochondrial proteins and contributes to mitochondrial heterogeneity. Cell Rep..

[40] Patel MS, Nemeria NS, Furey W, Jordan F (2014). The pyruvate dehydrogenase complexes: structure-based function and regulation. J. Biol. Chem..

[41] Green JD, Perham RN, Ullrich SJ, Appella E (1992). Conformational studies of the interdomain linker peptides in the dihydrolipoyl acetyltransferase component of the pyruvate dehydrogenase multienzyme complex of *Escherichia coli*. J. Biol. Chem..

[42] Bleile DM, Munk P, Oliver RM, Reed LJ (1979). Subunit structure of dihydrolipoyl transacetylase component of pyruvate dehydrogenase complex from *Escherichia coli*. Proc. Natl Acad. Sci. USA.

[43] Stephens PE, Darlison MG, Lewis HM, Guest JR (1983). The pyruvate dehydrogenase complex of *Escherichia coli* K12. Nucleotide sequence encoding the pyruvate dehydrogenase component. Eur. J. Biochem..

[44] Perham RN, Duckworth HW, Roberts GC (1981). Mobility of polypeptide chain in the pyruvate dehydrogenase complex revealed by proton NMR. Nature.

[45] Packman LC, Green B, Perham RN (1991). Lipoylation of the E2 components of the 2-oxo acid dehydrogenase multienzyme complexes of *Escherichia coli*. Biochem. J..

[46] Bevilacqua A, Li Z, Ho P-C (2022). Metabolic dynamics instruct CD8^+^ T‐cell differentiation and functions. Eur. J. Immunol..

[47] Elia I (2022). Tumor cells dictate anti-tumor immune responses by altering pyruvate utilization and succinate signaling in CD8^+^ T cells. Cell Metab..

[48] Pardee TS (2018). A phase I study of CPI-613 in combination with high dose cytarabine and mitoxantrone for relapsed or refractory acute myeloid leukemia.. Clin. Cancer Res..

[49] Philip PA (2019). A phase III open-label trial to evaluate efficacy and safety of CPI-613 plus modified FOLFIRINOX (mFFX) versus FOLFIRINOX (FFX) in patients with metastatic adenocarcinoma of the pancreas. Future Oncol..

[50] Dionisio KL (2018). The Chemical and Products Database, a resource for exposure-relevant data on chemicals in consumer products. Sci. Data.

[51] Russi AS (2018). Malignant brain tumors in patients with glutaric aciduria type I. Mol. Genet Metab..

[52] Carty SA (2018). The loss of TET2 promotes CD8^+^ T cell memory differentiation. J. Immunol..

[53] Fraietta JA (2018). Disruption of TET2 promotes the therapeutic efficacy of CD19-targeted T cells. Nature.

[54] Scharer CD, Bally APR, Gandham B, Boss JM (2017). Cutting edge: chromatin accessibility programs CD8 T cell memory. J. Immunol..

[55] Verma S (2022). NRF2 mediates melanoma addiction to GCDH by modulating apoptotic signalling.. Nat. Cell Biol..

[56] Bhatt DP (2022). Deglutarylation of glutaryl-CoA dehydrogenase by deacylating enzyme SIRT5 promotes lysine oxidation in mice. J. Biol. Chem..

[57] Cheng Y-M (2019). Lysine glutarylation in human sperm is associated with progressive motility. Hum. Reprod..

[58] Zhou L (2016). SIRT5 promotes IDH2 desuccinylation and G6PD deglutarylation to enhance cellular antioxidant defense. EMBO Rep..

[59] Bailey PSJ (2020). ABHD11 maintains 2-oxoglutarate metabolism by preserving functional lipoylation of the 2-oxoglutarate dehydrogenase complex. Nat. Commun..

[60] Seim GL (2023). Nitric oxide-driven modifications of lipoic arm inhibit α-ketoacid dehydrogenases. Nat. Chem. Biol..

[61] Mathias RA (2014). Sirtuin 4 is a lipoamidase regulating pyruvate dehydrogenase complex activity. Cell.

[62] Ryan HE, Lo J, Johnson RS (1998). HIF‐1α is required for solid tumor formation and embryonic vascularization. EMBO J..

[63] Sim J (2018). The factor inhibiting HIF asparaginyl hydroxylase regulates oxidative metabolism and accelerates metabolic adaptation to hypoxia. Cell Metab..

[64] Philip B (2014). A highly compact epitope-based marker/suicide gene for easier and safer T-cell therapy. Blood.

[65] Bofill-De Ros X, Gu S (2016). Guidelines for the optimal design of miRNA-based shRNAs. Methods.

[66] Fellmann C (2013). An optimized microRNA backbone for effective single-copy RNAi. Cell Rep..

[67] Moffat J (2006). A lentiviral RNAi library for human and mouse genes applied to an arrayed viral high-content screen. Cell.

[68] Hirsilä M, Koivunen P, Günzler V, Kivirikko KI, Myllyharju J (2003). Characterization of the human prolyl 4-hydroxylases that modify the hypoxia-inducible factor. J. Biol. Chem..

[69] Chakraborty AA (2019). Histone demethylase KDM6A directly senses oxygen to control chromatin and cell fate. Science.

