# Supplementary material for: Glutarate regulates T cell metabolism and anti-tumour immunity
Source: Nat Metab. 2023 Aug 21;5(10):1747–64. doi: 10.1038/s42255-023-00855-2 (PMC10590756; doi:10.1038/s42255-023-00855-2)
Supplement: Supplementary file 1 — Supplementary Tables 1–5. [file 42255_2023_855_MOESM1_ESM.pdf]

nature metabolism

Article https://doi.org/10.1038/s42255-023-00855-2

Glutarate regulates T cell metabolism and anti-tumour immunity

In the format provided by the authors and unedited

Supplementary Tables

Antibody	Fluorophore	Clone	Company	Catalogue Number	Dilution
CCR7	PE	3D12	BD Biosciences	552176	1:100
CCR7	PE/Cy7	3D12	BD Biosciences	557648	1:100
CD127	PE	A019D5	Biolegend	351303	1:200
CD127	APC	A019D5	Biolegend	351342	1:200
CD137	PE	4B4-1	Biolegend	309804	1:200
CD19	PE	4G7	Biolegend	392505	1:200
CD19	APC	4G7	Biolegend	392504	1:200
CD25	BV510	BC96	Biolegend	302639	1:200
CD27	PerCP/Cy5.5	M-T271	Biolegend	356408	1:200
CD3	BV510	HIT3a	BD Biosciences	564713	1:200
CD34 (RQR8)	AF488	QBEnd10	R&D	FAB7227G	1:100
CD44	FITC	BJ18	Biolegend	338803	1:200
CD45RA	BV650	HI101	Biolegend	304135	1:200
CD45RO	BUV496	UCHL1	BD Biosciences	749888	1:200
CD45RO	BV605	UCHL1	Biolegend	304238	1:200
CD62L	AF488	DREG-56	Biolegend	304816	1:200
CD62L	PerCP/Cy5.5	DREG-56	Biolegend	304824	1:200
CD8a	BUV395	RPA-T8	BD	563796	1:400
CD8a	AF700	HIT8a	Biolegend	300920	1:400
CD95	PerCP/Cy5.5	DX2	Biolegend	305630	1:200
GzmB	PerCP/Cy5.5	QA16A02	Biolegend	372211	1:200
GzmB	AF647	GB11	Biolegend	515405	1:200
LAG3 (CD223)	AF647	11C3C65	Biolegend	369304	1:200
Perforin	Pacific Blue	dG9	Biolegend	308117	1:200
Perforin	PerCP/Cy5.5	dG9	Biolegend	308114	1:200
TBET	PerCP/Cy5.5	4B10	Biolegend	644805	1:200

TBET	PE/Dazzle594	4B10	Biolegend	644828	1:200
TCF1	PE	S33-966	BD Biosciences	564217	1:200
TIGIT (VSTM3)	PE/Cy7	A15153G	Biolegend	372713	1:200
TIM3 (CD366)	BV605	F38-2E2	Biolegend	345017	1:200
TOX	eFluor660	TXRX10	eBiosciences	50-6502-82	1:200

Supplementary Table 1: List of human fluorochrome-labelled antibodies used for flow cytometry and sorting

Antibody	Fluorophore	Clone	Company	Catalogue Number	Dilution
CD11b	AF647	M1/70	Biolegend	101220	1:200
CD11b	AF488	M1/70	eBiosciences	53-0112-80	1:200
CD11b	AF700	M1/70	eBiosciences	56-0112-80	1:200
CD11c	PerCP/Cy5.5	N418	eBiosciences	45-0114-80	1:200
CD127	AF647	A7R34	Biolegend	135020	1:200
CD137	PE	17B5	Biolegend	106105	1:200
CD137	APC	17B5	Biolegend	106110	1:200
CD19	FITC	eBio1D3	eBiosciences	11-0193-82	1:200
CD19	PE	eBio1D3	eBiosciences	25-0193-82	1:200
CD19	APC	eBio1D3	eBiosciences	17-0193-82	1:200
CD25	PE	PC61.5	eBiosciences	12-0261-818	1:200
CD27	AF700	LG.3A10	Biolegend	124239	1:200
CD34	AF647	HM34	Biolegend	128605	1:200
CD3	PE/Dazzle	17A2	Biolegend	100245	1:200
CD4	BV421	GK1.5	Biolegend	100437	1:200
CD4	AF488	GK1.5	Biolegend	100423	1:200
CD4	BV650	RM4-5	Biolegend	100545	1:200
CD4	BV510	GK1.5	Biolegend	100449	1:200
CD44	PerCP/Cy5.5	IM7	Biolegend	103032	1:200
CD44	BUV496	IM7	BD Biosciences	741057	1:200

CD45.1	PerCP/Cy5.5	A20	eBiosciences	45-0453-82	1:200
CD45.1	PE	A20	Biolegend	110708	1:200
CD45.2	AF488	104	Biolegend	109815	1:200
CD45.2	BV421	104	BD Biosciences	562895	1:200
CD62L	AF488	MEL-14	Biolegend	104419	1:200
CD62L	PE	MEL-14	Biolegend	104408	1:200
CD62L	AF488	DREG-56	Biolegend	304816	1:200
CD62L	BUV737	MEL-14	BD Biosciences	612833	1:200
CD8a	BUV395	53-6.7	BD Biosciences	565968	1:400
CD8a	AF700	53-6.7	Biolegend	100730	1:400
CD8a	BV510	53-6.7	Biolegend	100751	1:400
CTLA4(CD152)	PerCP/Cy5.5	UC10-4B9	Biolegend	106315	1:200
GzmB	PerCP/Cy5.5	AD2	Biolegend	344013	1:200
LAG3(CD223)	PerCP/Cy5.5	C9B7W	Biolegend	125212	1:200
LAG3(CD223)	PE	C9B7W	BD Biosciences	552380	1:200
PD-1(CD279)	PerCP-eFluor™ 710	J43	Thermo Scientific	46-9985-82	1:200
Perforin	APC	eBioOMAK-D	Biolegend	17-9392-80	1:200
TBET	PerCP/Cy5.5	4B10	Biolegend	644805	1:200
TCF1	PE	S33-966	BD	564217	1:200
TIM3(CD366)	BV605	RMT3-23	Biolegend	119721	1:200
TIM3(CD366)	BV605	F38-2E2	Biolegend	345017	1:200
TOX	APC	TXRX10	eBiosciences	50-6502-82	1:200
TCRgd	BV421	RUO	Biolegend	118119	1:200
Ly6G	BV605	1A8	Biolegend	127639	1:200
Ly6C	PE/Cy7	HK1.4	Biolegend	128017	1:200
MHC II	BV650	M5/14.15.2	Biolegend	107641	1:200

NK1.1	PE	PK136	Biolegend	108726	1:200

Supplementary Table 2: List of mouse fluorochrome-labelled antibodies used for flow cytometry and sorting.

	Aminoacid sequence
**RQR8**	MGTSLLCWMALCLLGADHADACPYSNPSLCSGGGGSELPTQGTFSNVSTNVSPAKPTTTACPYSNPSLCSGGGGSPAPRPPTPAPTIASQPLSLRPEACRPAAGGAVHTRGLDFACDIYIWAPLAGTCGVLLLSLVITLYCNHRNRRRVCKCPRPVV
**T2A self-cleaving**	GSGEGRGSLLTCGDVEENPGP
**CD19 BBz CAR**	MALPVTALLLPLALLLHAARPDIQMTQTTSSLSASLGDRVTISCRASQDISKYLNWYQQKPDGTVKLLIYHTSRLHSGVPSRFSGSGSGTDYSLTISNLEQEDIATYFCQQGNTLPYTFGGGTKLEITGGGGSGGGGSGGGGSEVKLQESGPGLVAPSQSLSVTCTVSGVSLPDYGVSWIRQPPRKGLEWLGVIWGSETTYYNSALKSRLTIIKDNSKSQVFLKMNSLQTDDTAIYYCAKHYYYGGSYAMDYWGQGTSVTVSSTTTPAPRPPTPAPTIASQPLSLRPEACRPAAGGAVHTRGLDFACDIYIWAPLAGTCGVLLLSLVITLYCKRGRKKLLYIFKQPFMRPVQTTQEEDGCSCRFPEEEEGGCELRVKFSRSADAPAYKQGQNQLYNELNLGRREEYDVLDKRRGRDPEMGGKPRRKNPQEGLYNELQKDKMAEAYSEIGMKGERRRGKGHDGLYQGLSTATKDTYDALHMQALPPR
**HER2 (4D5) BBz CAR**	MALPVTALLLPLALLLHAARPMDFQVQIFSFLLISASVIMSRGDIQMTQSPSSLSASVGDRVTITCRASQDVNTAVAWYQQKPGKAPKLLIYSASFLYSGVPSRFSGSRSGTDFTLTISSLQPEDFATYYCQQHYTTPPTFGQGTKVEIKRTGSTSGSGKPGSGEGSEVQLVESGGGLVQPGGSLRLSCAASGFNIKDTYIHWVRQAPGKGLEWVARIYPTNGYTRYADSVKGRFTISADTSKNTAYLQMNSLRAEDTAVYYCSRWGGDGFYAMDVWGQGTLVTVSSTTTPAPRPPTPAPTIASQPLSLRPEACRPAAGGAVHTRGLDFACDIYIWAPLAGTCGVLLLSLVITLYCKRGRKKLLYIFKQPFMRPVQTTQEEDGCSCRFPEEEEGGCELRVKFSRSADAPAYKQGQNQLYNELNLGRREEYDVLDKRRGRDPEMGGKPRRKNPQEGLYNELQKDKMAEAYSEIGMKGERRRGKGHDGLYQGLSTATKDTYDALHMQALPPR
**GCDH**	MALRGVSVRLLSRGPGLHVLRTWVSSAAQTEKGGRTQSQLAKSSRPEFDWQDPLVLEEQLTTDEILIRDTFRTYCQERLMPRILLANRNEVFHREIISEMGELGVLGPTIKGYGCAGVSSVAYGLLARELERVDSGYRSAMSVQSSLVMHPIYAYGSEEQRQKYLPQLAKGELLGCFGLTEPNSGSDPSSMETRAHYNSSNKSYTLNGTKTWITNSPMADLFVVWARCEDGCIRGFLLEKGMRGLSAPRIQGKFSLRASATGMIIMDGVEVPEENVLPGASSLGGPFGCLNNARYGIAWGVLGASEFCLHTARQYALDRMQFGVPLARNQLIQKKLADMLTEITLGLHACLQLGRLKDQDKAAPEMVSLLKRNNCGKALDIARQARDMLGGNGISDEYHVIRHAMNLEAVNTYEGTHDIHALILGRAITGIQAFTASK
	**DNA sequence**
**miRNA-embedded shNTC**	TGACGTACGGCGCGCGCCTCGACTAGGGATAACAGGGTAATTGTTTGAATGAGGCTTCAGTACTTTACAGAATCGTTGCCTGCACATCTTGGAAACACTTGCTGGGATTACTTCGACTTCTTAACCCAACAGAAGGCTCGAGAAGGTATATTGCTGTTGACAGTGAGCGCAGGAATTATAATGCTTATCTATAGTGAAGCCACAGATGTATAGATAAGCATTATAATTCCTATGCCTACTGCCTCGGACTTCAAGGGGCTAGAATTCGAGCAATTATCTTGTTTACTAAAACTGAATACCTTGCTATCTCTTTGATACATTTTTACAAAGCTGAATTAAAATGGTATAAATTAAATCACTTTTTTCAATTGACGCGTAATTCTACCTGA
**miRNA-embedded GCDH**	TGACGTACGGCGCGCGCCTCGACTAGGGATAACAGGGTAATTGTTTGAATGAGGCTTCAGTACTTTACAGAATCGTTGCCTGCACATCTTGGAAACACTTGCTGGGATTACTTCGACTTCTTAACCCAACAGAAGGCTCGAGAAGGTATATTGCTGTTGACAGTGAGCGCATGGGATTTCTGACGAGTATCTAGTGAAGCCACAGATGTAGATACTCGTCAGAAATCCCATATGCCTACTGCCTCGGACTTCAAGGGGCTAGAATTCGAGCAATTATCTTGTTTACTAAAACTGAATACCTTGCTATCTCTTTGATACATTTTTACAAAGCTGAATTAAAATGGTATAAATTAAATCACTTTTTTCAATTGACGCGTAATTCTACCTGA

Supplementary Table 3: List of protein sequences and DNA sequences corresponding to miRNA-embedded shRNAs used in viral vectors.

MRM function	Compound	Parent ion (m/z)	Ion 1 (quantifier, m/z)	Collision energy (eV)	Ion 2 (qualifier, m/z)	Collision energy (eV)	Rt (min)	Polarity
**1**	DEG	189,1	143,1	7	115,1	14	0.93	POS
**2**	DEG-$^{13}C_5$	194,1	148,1	7	120,1	14	0.93	POS
**3**	DEG-$^{2}H_4$	193,1	147,1	7	118,7	14	0.93	POS
**4**	GA	131,1	87,1	10	69,1	13	2.08	NEG
**5**	GA-$^{2}H_4$	135,1	91,1	10	72,2	13	2.08	NEG
**6**	GA-$^{13}C_5$	136,1	91,1	10	73.1	13	2.08	NEG
**7**	2-OH-GA	147,1	129,1	10	85,1	14	3.29	NEG
**8**	2-OH-GA-$^{13}C_5$	152,1	134,1	10	89.1	14	3.29	NEG
**9**	2-OH-GA-$^{2}H_4$	150,1	132,1	10	87.1	14	3.29	NEG
**10**	CHES	206,1	107,1	21	79,1	25	4.32	NEG

Supplementary Table 4: Mass transitions and parameters

Gene Symbol	Forward Primer (5' to 3')	Reverse Primer (5'-3')	Reverse Primer (5’ to 3’)
BNIP3	CAGTCTGAGGAAGATGATATTG	BNIP3	GTGTTTAAAGAGGAACTCCTTG
PDHK1	AAGAGTGCTGATTGAGTAAC	PDHK1	ATGATGTCATTCCCACAATG
CA9	GTGCCTATGAGCAGTTGCTGTC	CA9	AAGTAGCGGCTGAAGTCAGAGG
GLUT1	ATACTCATGACCATCGCGCTAG	GLUT1	AAAGAAGGCCACAAAGCCAAAG
CPT1A	GATCCTGGACAATACCTCGGAG	CPT1A	CTCCACAGCATCAAGAGACTGC
FASN	CACACACGATGGACCCTCAG	FASN	AATCTGGGTTGATGCCTCCG
FABP2	CACACACGATGGACCCTCAG	FABP2	AATCTGGGTTGATGCCTCCG

Supplementary Table 5: List of primers used
