# Supplementary material for: Glutarate regulates T cell metabolism and anti-tumour immunity
Source: Nat Metab. 2023 Aug 21;5(10):1747–64. doi: 10.1038/s42255-023-00855-2 (PMC10590756; doi:10.1038/s42255-023-00855-2)
Supplement: Supplementary file 2 — Reporting Summary [file 42255_2023_855_MOESM2_ESM.pdf]

nature portfolio

Corresponding author(s): Randall Johnson

Last updated by author(s): 17/06/2023

Reporting Summary

Nature Portfolio wishes to improve the reproducibility of the work that we publish. This form provides structure for consistency and transparency in reporting. For further information on Nature Portfolio policies, see our Editorial Policies and the Editorial Policy Checklist.

Statistics

For all statistical analyses, confirm that the following items are present in the figure legend, table legend, main text, or Methods section.

n/a	Confirmed	
☐	☒	The exact sample size (*n*) for each experimental group/condition, given as a discrete number and unit of measurement
☐	☒	A statement on whether measurements were taken from distinct samples or whether the same sample was measured repeatedly
☐	☒	The statistical test(s) used AND whether they are one- or two-sided<br>*Only common tests should be described solely by name; describe more complex techniques in the Methods section.*
☐	☒	A description of all covariates tested
☐	☒	A description of any assumptions or corrections, such as tests of normality and adjustment for multiple comparisons
☐	☒	A full description of the statistical parameters including central tendency (e.g. means) or other basic estimates (e.g. regression coefficient) AND variation (e.g. standard deviation) or associated estimates of uncertainty (e.g. confidence intervals)
☐	☒	For null hypothesis testing, the test statistic (e.g. *F*, *t*, *r*) with confidence intervals, effect sizes, degrees of freedom and *P* value noted<br>*Give P values as exact values whenever suitable.*
☒	☐	For Bayesian analysis, information on the choice of priors and Markov chain Monte Carlo settings
☒	☐	For hierarchical and complex designs, identification of the appropriate level for tests and full reporting of outcomes
☒	☐	Estimates of effect sizes (e.g. Cohen's *d*, Pearson's *r*), indicating how they were calculated

Our web collection on statistics for biologists contains articles on many of the points above.

Software and code

Policy information about availability of computer code

Data collection	Aurora (Cytek Biosciences); Aria III (BD Biosciences); microplate reader (Sunrise, Tecan Austria GmbH); Waters Acquity UPLC system coupled to a Xevo-TQ-S mass spectrometer (Waters, Milford, MA, USA); StepOnePlus system (Applied Biosystems); iBrightCL1000 (Thermo Fisher); Q Exactive HF hybrid Orbitrap mass spectrometer (Thermo Fisher Scientific) ; an Orbitrap Fusion Lumos coupled to an Ultimate 3000 RSLC nano UHPLC equipped with a 100 µm ID x 2 cm Acclaim PepMap Precolumn (Thermo Fisher Scientific) ; Seahorse XFe bioanalyser (Agilent) ; on FLUROstar Omega (BMG Labtech)
Data analysis	FlowJo_V10.7.2; Mascot Server v.2.5.1 (Matrix Science Ltd., UK) ; PEAKS Studio (version 8.0, Bioinformatics Solutions Inc.) ; Prism 9 software (GraphPad) ; iBright Analysis Software_V4.0.1 ; ImageJ;

For manuscripts utilizing custom algorithms or software that are central to the research but not yet described in published literature, software must be made available to editors and reviewers. We strongly encourage code deposition in a community repository (e.g. GitHub). See the Nature Portfolio guidelines for submitting code & software for further information.

Data

Policy information about availability of data

All manuscripts must include a data availability statement. This statement should provide the following information, where applicable:

- Accession codes, unique identifiers, or web links for publicly available datasets
- A description of any restrictions on data availability
- For clinical datasets or third party data, please ensure that the statement adheres to our policy

All data generated or analysed during this study are included in the published article and its supplementary information files. The data that support the findings of this study are available from the corresponding author upon reasonable request. Source data are provided with this paper

Human research participants

Policy information about studies involving human research participants and Sex and Gender in Research.

Reporting on sex and gender	Sex and gender of healthy volunteer blood donors was not obtained as per ethical approval requirements.
Population characteristics	Human peripheral blood mononuclear cells (PBMCs) were obtained from National Health Service (NHS) Blood and Transplant (NHSBT: Addenbrooke's Hospital, Cambridge, United Kingdom) or Karolinska Hospital Service, Sweden. All volunteers were healthy as defined by national blood donation standards and over the age of 18.
Recruitment	Human peripheral blood mononuclear cells (PBMCs) were obtained from National Health Service (NHS) Blood and Transplant (NHSBT: Addenbrooke's Hospital, Cambridge, United Kingdom) or Karolinska Hospital Service, Sweden, from healthy volunteers between the ages of 20 and 40.
Ethics oversight	Ethical approval was obtained from the East of England-Cambridge Central Research Ethics Committee (06/Q0108/281) .

Note that full information on the approval of the study protocol must also be provided in the manuscript.

Field-specific reporting

Please select the one below that is the best fit for your research. If you are not sure, read the appropriate sections before making your selection.

☒ Life sciences ☐ Behavioural & social sciences ☐ Ecological, evolutionary & environmental sciences

For a reference copy of the document with all sections, see nature.com/documents/nr-reporting-summary-flat.pdf

Life sciences study design

All studies must disclose on these points even when the disclosure is negative.

Sample size	Power calculations were carried out where effect size could be estimated.
Data exclusions	Mice who did not grow tumors were excluded from the study. For in-vitro studies, data point identified as an outlier by independent analysis using Prism Software were excluded.
Replication	All experiments were replicated in at least three independent studies with the following exceptions:<br>1. Extended Data Fig. 1m: This was repeated independently 3 times but an error was made with one sample during the final derivation for mass spectrometry. Thus, only two independent results are reported.<br>2. Fig 5f: This experiment was performed independently twice with the same result observed both times. It was not performed a third time due to the significant cost involved and the desire to keep use of animals to a minimum.<br>3. Fig. 5m and Extended Data Fig. 5g-i: This experiment was performed independently once with at least 8 biological replicates per group (i.e. animals). Tumor growth and blood sampling was performed in at least 3 independent studies however, terminal experiments (tumor, spleen and lymph node analysis) was only performed once. In Fig. 1m all animals displayed the same trend. Power calculations were carried out to estimate effect size.
Randomization	Randomization for in vitro studies was not possible as groups were separated based on treatment. Randomization was preformed for in vivo studies. In animal studies, mice were randomly assigned groups using an Online random number generator.
Blinding	In vitro studies were not blinded as they all required specific culture conditions for treatment groups. Blinding in the animal experiments was not possible as animals were separated based on treatment type. Analysis of blood and tissues from animal experiments was blinded.

Behavioural & social sciences study design

All studies must disclose on these points even when the disclosure is negative.

Study description	*Briefly describe the study type including whether data are quantitative, qualitative, or mixed-methods (e.g. qualitative cross-sectional, quantitative experimental, mixed-methods case study).*
Research sample	*State the research sample (e.g. Harvard university undergraduates, villagers in rural India) and provide relevant demographic information (e.g. age, sex) and indicate whether the sample is representative. Provide a rationale for the study sample chosen. For studies involving existing datasets, please describe the dataset and source.*
Sampling strategy	*Describe the sampling procedure (e.g. random, snowball, stratified, convenience). Describe the statistical methods that were used to predetermine sample size OR if no sample-size calculation was performed, describe how sample sizes were chosen and provide a rationale for why these sample sizes are sufficient. For qualitative data, please indicate whether data saturation was considered, and what criteria were used to decide that no further sampling was needed.*
Data collection	*Provide details about the data collection procedure, including the instruments or devices used to record the data (e.g. pen and paper, computer, eye tracker, video or audio equipment) whether anyone was present besides the participant(s) and the researcher, and whether the researcher was blind to experimental condition and/or the study hypothesis during data collection.*
Timing	*Indicate the start and stop dates of data collection. If there is a gap between collection periods, state the dates for each sample cohort.*
Data exclusions	*If no data were excluded from the analyses, state so OR if data were excluded, provide the exact number of exclusions and the rationale behind them, indicating whether exclusion criteria were pre-established.*
Non-participation	*State how many participants dropped out/declined participation and the reason(s) given OR provide response rate OR state that no participants dropped out/declined participation.*
Randomization	*If participants were not allocated into experimental groups, state so OR describe how participants were allocated to groups, and if allocation was not random, describe how covariates were controlled.*

Ecological, evolutionary & environmental sciences study design

All studies must disclose on these points even when the disclosure is negative.

Study description	*Briefly describe the study. For quantitative data include treatment factors and interactions, design structure (e.g. factorial, nested, hierarchical), nature and number of experimental units and replicates.*
Research sample	*Describe the research sample (e.g. a group of tagged Passer domesticus, all Stenocereus thurberi within Organ Pipe Cactus National Monument), and provide a rationale for the sample choice. When relevant, describe the organism taxa, source, sex, age range and any manipulations. State what population the sample is meant to represent when applicable. For studies involving existing datasets, describe the data and its source.*
Sampling strategy	*Note the sampling procedure. Describe the statistical methods that were used to predetermine sample size OR if no sample-size calculation was performed, describe how sample sizes were chosen and provide a rationale for why these sample sizes are sufficient.*
Data collection	*Describe the data collection procedure, including who recorded the data and how.*
Timing and spatial scale	*Indicate the start and stop dates of data collection, noting the frequency and periodicity of sampling and providing a rationale for these choices. If there is a gap between collection periods, state the dates for each sample cohort. Specify the spatial scale from which the data are taken*
Data exclusions	*If no data were excluded from the analyses, state so OR if data were excluded, describe the exclusions and the rationale behind them, indicating whether exclusion criteria were pre-established.*
Reproducibility	*Describe the measures taken to verify the reproducibility of experimental findings. For each experiment, note whether any attempts to repeat the experiment failed OR state that all attempts to repeat the experiment were successful.*
Randomization	*Describe how samples/organisms/participants were allocated into groups. If allocation was not random, describe how covariates were controlled. If this is not relevant to your study, explain why.*
Blinding	*Describe the extent of blinding used during data acquisition and analysis. If blinding was not possible, describe why OR explain why blinding was not relevant to your study.*

Did the study involve field work? ☐ Yes ☒ No

Reporting for specific materials, systems and methods

We require information from authors about some types of materials, experimental systems and methods used in many studies. Here, indicate whether each material, system or method listed is relevant to your study. If you are not sure if a list item applies to your research, read the appropriate section before selecting a response.

Materials & experimental systems

n/a	Involved in the study
☐	☒ Antibodies
☐	☒ Eukaryotic cell lines
☒	☐ Palaeontology and archaeology
☐	☒ Animals and other organisms
☒	☐ Clinical data
☒	☐ Dual use research of concern

Methods

n/a	Involved in the study
☒	☐ ChIP-seq
☐	☒ Flow cytometry
☒	☐ MRI-based neuroimaging

Antibodies

Antibodies used

Antibody Company Catalogue Number Dilution
anti-5hmC Active Motif 10013602
anti-PDHK1 cell signalling 3820 1:1000
anti-PPIB cell signalling 43603 1:1000
anti-Phospho-PDH a1 cell signalling 37115 1:1000
anti-SIRT5 cell signalling 8779S 1:1000
anti-H3 cell signalling 4499 1:1000
anti-H3K4me2 cell signalling 9725 1:1000
anti-H3K4me3 cell signalling 9751 1:1000
anti-H3K9me2 cell signalling 4658 1:1000
anti-H3K9me3 cell signalling 13969 1:1000
anti-H3K27me2 cell signalling 9728 1:1000
anti-H3K27me3 cell signalling 9733 1:1000
anti-H3K36me2 cell signalling 2901 1:1000
anti-H3K36me3 cell signalling 4909 1:1000
anti-H3K79me2 cell signalling 5427 1:1000
anti-H3K79me3 cell signalling 4360 1:1000
anti-b-actin cell signalling 12262S 1:1000
PDHc Thermo Scientific 456799 1:1000
GCDH Thermo Scientific PA5-60294 1:1000
anti-lipoic acid Sigma 437695 1:1000
pan anti-glutarylysine PTM Biolabs PTM-1151 1:1000
Anti-OGDH cell signalling 26865S 1:1000
Anti-DLAT cell signalling 12362S 1:1000
Rabbit IgG HPR R&D HAF008 1:5000
Mouse IgG HPR R&D HAF009 1:1000

Antibody Fluorophore Clone Company Catalogue Number Dilution
CCR7 PE 3D12 BD Biosciences 552176
1:100
CCR7 PE/Cy7 3D12 BD Biosciences 557648
1:100
CD127 PE A019D5 Biolegend 351303 1:200
CD127 APC A019D5 Biolegend 351342 1:200
CD137 PE 4B4-1 Biolegend 309804 1:200
CD19 PE 4G7 Biolegend 392505 1:200
CD19 APC 4G7 Biolegend 392504 1:200
CD25 BV510 BC96 Biolegend 302639 1:200
CD27 PerCP/Cy5.5 M-T271 Biolegend 356408 1:200
CD3 BV510 HIT3a BD Biosciences 564713
1:200
CD34 (RQR8) AF488 QBEnd10 R&D FAB7227G
1:100
CD44 FITC BJ18 Biolegend 338803 1:200
CD45RA BV650 HI101 Biolegend 304135 1:200
CD45RO BUV496 UCHL1 BD Biosciences 749888
1:200
CD45RO BV605 UCHL1 Biolegend 304238 1:200
CD62L AF488 DREG-56 Biolegend 304816 1:200
CD62L PerCP/Cy5.5 DREG-56 Biolegend 304824 1:200
CD8a BUV395 RPA-T8 BD 563796

1:400
CD8a AF700 HIT8a Biolegend 300920 1:400
CD95 PerCP/Cy5.5 DX2 Biolegend 305630 1:200
GzmB PerCP/Cy5.5 QA16A02 Biolegend 372211 1:200
GzmB AF647 GB11 Biolegend 515405 1:200
LAG3 (CD223) AF647 11C3C65 Biolegend 369304 1:200
Perforin Pacific Blue dG9 Biolegend 308117
1:200
Perforin PerCP/Cy5.5 dG9 Biolegend 308114 1:200
TBET PerCP/Cy5.5 4B10 Biolegend 644805 1:200
TBET PE/Dazzle594 4B10 Biolegend 644828
1:200
TCF1 PE S33-966 BD Biosciences 564217
1:200
TIGIT (VSTM3) PE/Cy7 A15153G Biolegend 372713 1:200
TIM3 (CD366) BV605 F38-2E2 Biolegend 345017 1:200
TOX eFluor660 TXRX10 eBiosciences 50-6502-82 1:200
Antibody Fluorophore Clone Company Catalogue Number Dilution
CD11b AF647 M1/70 Biolegend 101220 1:200
CD11b AF488 M1/70 eBiosciences 53-0112-80 1:200
CD11b AF700 M1/70 eBiosciences 56-0112-80 1:200
CD11c PerCP/Cy5.5 N418 eBiosciences 45-0114-80 1:200
CD127 AF647 A7R34 Biolegend 135020 1:200
CD137 PE 17B5 Biolegend 106105 1:200
CD137 APC 17B5 Biolegend 106110 1:200
CD19 FITC eBio1D3 eBiosciences 11-0193-82 1:200
CD19 PE eBio1D3 eBiosciences 25-0193-82 1:200
CD19 APC eBio1D3 eBiosciences 17-0193-82 1:200
CD25 PE PC61.5 eBiosciences 12-0261-818 1:200
CD27 AF700 LG.3A10 Biolegend 124239 1:200
CD34 AF647 HM34 Biolegend 128605 1:200
CD3 PE/Dazzle 17A2 Biolegend 100245 1:200
CD4 BV421 GK1.5 Biolegend 100437 1:200
CD4 AF488 GK1.5 Biolegend 100423 1:200
CD4 BV650 RM4-5 Biolegend 100545 1:200
CD4 BV510 GK1.5 Biolegend 100449 1:200
CD44 PerCP/Cy5.5 IM7 Biolegend 103032 1:200
CD44 BUV496 IM7 BD Biosciences 741057 1:200
CD45.1 PerCP/Cy5.5 A20 eBiosciences 45-0453-82 1:200
CD45.1 PE A20 Biolegend 110708 1:200
CD45.2 AF488 104 Biolegend 109815 1:200
CD45.2 BV421 104 BD Biosciences 562895 1:200
CD62L AF488 MEL-14 Biolegend 104419 1:200
CD62L PE MEL-14 Biolegend 104408 1:200
CD62L AF488 DREG-56 Biolegend 304816 1:200
CD62L BUV737 MEL-14 BD Biosciences 612833 1:200
CD8a BUV395 53-6.7 BD Biosciences 565968 1:400
CD8a AF700 53-6.7 Biolegend 100730 1:400
CD8a BV510 53-6.7 Biolegend 100751 1:400
CTLA4(CD152) PerCP/Cy5.5 UC10-4B9 Biolegend 106315 1:200
GzmB PerCP/Cy5.5 AD2 Biolegend 344013 1:200
LAG3(CD223) PerCP/Cy5.5 C9B7W Biolegend 125212 1:200
LAG3(CD223) PE C9B7W BD Biosciences 552380 1:200
PD-1(CD279) PerCP-eFluorTM 710 J43 Thermo Scientific 46-9985-82 1:200
Perforin APC eBioOMAK-D Biolegend 17-9392-80 1:200
TBET PerCP/Cy5.5 4B10 Biolegend 644805 1:200
TCF1 PE S33-966 BD 564217
1:200
TIM3(CD366) BV605 RMT3-23 Biolegend 119721 1:200
TIM3(CD366) BV605 F38-2E2 Biolegend 345017 1:200
TOX APC TXRX10 eBiosciences 50-6502-82 1:200
TCRgd BV421 RUO Biolegend 118119 1:200
Ly6G BV605 1A8 Biolegend 127639 1:200
Ly6C PE/Cy7 HK1.4 Biolegend 128017 1:200
MHC II BV650 M5/14.15.2 Biolegend 107641 1:200
NK1.1 PE PK136 Biolegend 108726 1:200

Validation

All antibodies were purchased from commercial companies as indicated. Antibodies were validated by sited companies. No unvalidated antibody was used in this study.

Eukaryotic cell lines

Policy information about cell lines and Sex and Gender in Research

Cell line source(s)	B16-F10 ATCC (CEL-6475); HEK293T from Takara (632180) ; SKOV3 from ATCC (HTB-77); Raji-GFP-Luc cells from Biocytogen (B-HCL-010) ; Jurkat from ATCC (TIB-152) ; RAW 264.7 rom ATCC (TIB-71); HeLa gift from Prof. Paul Lehner (University of Cambridge);
Authentication	B16-F10, SKOV3 and Raji cells were authenticized using cell surface antigen expression RAW,and HEK293 cells were not authenticized further than ensuring they looked and grew according to published data. HeLa cells were authenticated by short tandem repeat profiling (Eurofins Genomics).
Mycoplasma contamination	Cells tested negative for mycoplasma contamination.
Commonly misidentified lines (See ICLAC register)	The following cell lines used in this study are reported by the International Cell Line Authentication Committee (https://iclac.org/databases/cross-contaminations/) as known misidentified cell lines:<br>1. HEK293T<br>2. Raji<br>3. Jurkat<br>4. Raw 264.7<br>5. HeLa<br><br>Each of these cell lines (except for HeLa and Raw 264.7 cells) were commercially purchased for the purpose of this study. Authenticity of each cell line was confirmed by the supplier prior to purchase. Supplier information is provided in the methods section.<br><br>HeLa cells were authenticated by short tandem repeat profiling (Eurofins Genomics).

Palaeontology and Archaeology

Specimen provenance	*Provide provenance information for specimens and describe permits that were obtained for the work (including the name of the issuing authority, the date of issue, and any identifying information). Permits should encompass collection and, where applicable, export.*
Specimen deposition	*Indicate where the specimens have been deposited to permit free access by other researchers.*
Dating methods	*If new dates are provided, describe how they were obtained (e.g. collection, storage, sample pretreatment and measurement), where they were obtained (i.e. lab name), the calibration program and the protocol for quality assurance OR state that no new dates are provided.*

☐ Tick this box to confirm that the raw and calibrated dates are available in the paper or in Supplementary Information.

Ethics oversight	*Identify the organization(s) that approved or provided guidance on the study protocol, OR state that no ethical approval or guidance was required and explain why not.*

Note that full information on the approval of the study protocol must also be provided in the manuscript.

Animals and other research organisms

Policy information about studies involving animals; ARRIVE guidelines recommended for reporting animal research, and Sex and Gender in Research

Laboratory animals	C57BL/6J animals (632, Charles River), were used in in vitro assays and in orthotopic tumor growth and infiltration experiments (male and female mice used; 8-12 weeks of age). NOD.Cg-PrkdcscidIl2rgtm1Wjl/SzJ mice (005557, The Jackson Laboratory) were used in human CAR-T cell experiments. Donor TCR-transgenic OT1 mice (003831, The Jackson Laboratory) were crossed with mice bearing the CD45.1 congenic marker (002014, The Jackson Laboratory). Targeted deletion of HIF1 in T cells was achieved by crossing homozygous mice carrying loxP-flanked alleles Hif1 61 into a mouse strain of cre recombinase expression driven by the distal promoter of the lymphocyte-specific Lck gene (012837, The Jackson Laboratory). All the experiments were performed with age and sex matched cre negative controls. TCR-transgenic OT1 mice and mice with targeted deletion of HIF1 in T cells were bred and housed in specific pathogen-free conditions in accordance with the regional animal ethics Committee of Northern Stockholm, Sweden. All animal experiments were performed in accordance with the ethical regulation of the UK Home Office and the University of Cambridge and/or the regional animal ethics Committee of Northern Stockholm, Sweden.<br><br>Housing conditions were as follows: 12 hour light cycle with gradual change. Temperature; 22-23°C. Humidity; 50%.
Wild animals	No wild animals were used in this study.
Reporting on sex	Female and male mice were used in in vitro and in vivo studies. No sex or gender differences were observed.

Field-collected samples: No filed collected samples were used in this study.

Ethics oversight: UK Home Office and regional animal ethics Committee of Northern Stockholm, Sweden

Note that full information on the approval of the study protocol must also be provided in the manuscript.

Clinical data

Policy information about clinical studies

All manuscripts should comply with the ICMJE guidelines for publication of clinical research and a completed CONSORT checklist must be included with all submissions.

Clinical trial registration: *Provide the trial registration number from ClinicalTrials.gov or an equivalent agency.*

Study protocol: *Note where the full trial protocol can be accessed OR if not available, explain why.*

Data collection: *Describe the settings and locales of data collection, noting the time periods of recruitment and data collection.*

Outcomes: *Describe how you pre-defined primary and secondary outcome measures and how you assessed these measures.*

Dual use research of concern

Policy information about dual use research of concern

Hazards

Could the accidental, deliberate or reckless misuse of agents or technologies generated in the work, or the application of information presented in the manuscript, pose a threat to:

No	Yes	
☒	☐	Public health
☒	☐	National security
☒	☐	Crops and/or livestock
☒	☐	Ecosystems
☒	☐	Any other significant area

Experiments of concern

Does the work involve any of these experiments of concern:

No	Yes	
☒	☐	Demonstrate how to render a vaccine ineffective
☒	☐	Confer resistance to therapeutically useful antibiotics or antiviral agents
☒	☐	Enhance the virulence of a pathogen or render a nonpathogen virulent
☒	☐	Increase transmissibility of a pathogen
☒	☐	Alter the host range of a pathogen
☒	☐	Enable evasion of diagnostic/detection modalities
☒	☐	Enable the weaponization of a biological agent or toxin
☒	☐	Any other potentially harmful combination of experiments and agents

ChIP-seq

Data deposition

☐ Confirm that both raw and final processed data have been deposited in a public database such as GEO.

☐ Confirm that you have deposited or provided access to graph files (e.g. BED files) for the called peaks.

Data access links
May remain private before publication.: *For "Initial submission" or "Revised version" documents, provide reviewer access links. For your "Final submission" document, provide a link to the deposited data.*

Files in database submission: *Provide a list of all files available in the database submission.*

Genome browser session
(e.g. UCSC): *Provide a link to an anonymized genome browser session for "Initial submission" and "Revised version" documents only, to enable peer review. Write "no longer applicable" for "Final submission" documents.*

Methodology

Replicates

Describe the experimental replicates, specifying number, type and replicate agreement.

Sequencing depth

Describe the sequencing depth for each experiment, providing the total number of reads, uniquely mapped reads, length of reads and whether they were paired- or single-end.

Antibodies

Describe the antibodies used for the ChIP-seq experiments; as applicable, provide supplier name, catalog number, clone name, and lot number.

Peak calling parameters

Specify the command line program and parameters used for read mapping and peak calling, including the ChIP, control and index files used.

Data quality

Describe the methods used to ensure data quality in full detail, including how many peaks are at FDR 5% and above 5-fold enrichment.

Software

Describe the software used to collect and analyze the ChIP-seq data. For custom code that has been deposited into a community repository, provide accession details.

Flow Cytometry

Plots

Confirm that:

☒ The axis labels state the marker and fluorochrome used (e.g. CD4-FITC).

☒ The axis scales are clearly visible. Include numbers along axes only for bottom left plot of group (a 'group' is an analysis of identical markers).

☒ All plots are contour plots with outliers or pseudocolor plots.

☒ A numerical value for number of cells or percentage (with statistics) is provided.

Methodology

Sample preparation

Single cell suspensions were stained with Near-IR Dead Cell Stain Kit (10119, Thermo Fisher) followed by surface and intracellular staining with fluorochrome-labelled antibodies (Supplementary Table 1). Staining of cytoplasmic and nuclear antigens was performed using the Fixation/Permeabilization kit (554714, BD Biosciences) and the Transcription Factor buffer set (562725, BD Biosciences), respectively. For proliferation assays, cells were loaded with Cell Trace Violet (C34557, Thermo Fisher) according to manufacturer's instructions. Samples were acquired on an Aurora (Cytek Biosciences).
For 5hmC staining, cells were stained for surface antigens as above and then fixed and permeabilised with the Transcription Factor buffer set (562725, BD Biosciences). Next cells were incubated with 4M HCL for 10 min at room temperature. The cells were then thoroughly washed and incubated in blocking buffer (0.1% PBS-Triton, 5% FBS) for 30min at 4oC. The cells were then incubated with primary anti-5hmC (10013602; Active Motif) overnight at 4oC and the day after with secondary antibody for 1 hr at room temperature. Flow cytometry was then performed as explained above.
Single cell suspensions were stained with Near-IR Dead Cell Stain Kit (10119, Thermo Fisher) followed by surface and intracellular staining with fluorochrome-labelled antibodies (Supplementary Table 1). Staining of cytoplasmic and nuclear antigens was performed using the Fixation/Permeabilization kit (554714, BD Biosciences) and the Transcription Factor buffer set (562725, BD Biosciences), respectively. For proliferation assays, cells were loaded with Cell Trace Violet (C34557, Thermo Fisher) according to manufacturer's instructions. Samples were acquired on an Aurora (Cytek Biosciences).
For 5hmC staining, cells were stained for surface antigens as above and then fixed and permeabilised with the Transcription Factor buffer set (562725, BD Biosciences). Next cells were incubated with 4M HCL for 10 min at room temperature. The cells were then thoroughly washed and incubated in blocking buffer (0.1% PBS-Triton, 5% FBS) for 30min at 4oC. The cells were then incubated with primary anti-5hmC (10013602; Active Motif) overnight at 4oC and the day after with secondary antibody for 1 hr at room temperature. Flow cytometry was then performed as explained above.
Single cell suspensions were stained with Near-IR Dead Cell Stain Kit (10119, Thermo Fisher) followed by surface and intracellular staining with fluorochrome-labelled antibodies (Supplementary Table 1). Staining of cytoplasmic and nuclear antigens was performed using the Fixation/Permeabilization kit (554714, BD Biosciences) and the Transcription Factor buffer set (562725, BD Biosciences), respectively. For proliferation assays, cells were loaded with Cell Trace Violet (C34557, Thermo Fisher) according to manufacturer's instructions. Samples were acquired on an Aurora (Cytek Biosciences).
For 5hmC staining, cells were stained for surface antigens as above and then fixed and permeabilised with the Transcription Factor buffer set (562725, BD Biosciences). Next cells were incubated with 4M HCL for 10 min at room temperature. The cells were then thoroughly washed and incubated in blocking buffer (0.1% PBS-Triton, 5% FBS) for 30min at 4oC. The cells were then incubated with primary anti-5hmC (10013602; Active Motif) overnight at 4oC and the day after with secondary antibody for 1 hr at room temperature. Flow cytometry was then performed as explained above.
Single cell suspensions were stained with Near-IR Dead Cell Stain Kit (10119, Thermo Fisher) followed by surface and intracellular staining with fluorochrome-labelled antibodies (Supplementary Table 1). Staining of cytoplasmic and nuclear antigens was performed using the Fixation/Permeabilization kit (554714, BD Biosciences) and the Transcription Factor buffer set (562725, BD Biosciences), respectively. For proliferation assays, cells were loaded with Cell Trace Violet (C34557, Thermo Fisher) according to manufacturer's instructions. Samples were acquired on an Aurora (Cytek Biosciences).

Instrument

Aurora (Cytek Biosciences).

Software

FlowJo

Cell population abundance	evenets gated on live CD8+ cells prior to determining expression of any markers
Gating strategy	FSC/SSC; FSC-A/FSC-H

☐ Tick this box to confirm that a figure exemplifying the gating strategy is provided in the Supplementary Information.

Magnetic resonance imaging

Experimental design

Design type	*Indicate task or resting state; event-related or block design.*
Design specifications	*Specify the number of blocks, trials or experimental units per session and/or subject, and specify the length of each trial or block (if trials are blocked) and interval between trials.*
Behavioral performance measures	*State number and/or type of variables recorded (e.g. correct button press, response time) and what statistics were used to establish that the subjects were performing the task as expected (e.g. mean, range, and/or standard deviation across subjects).*

Acquisition

Imaging type(s)	*Specify: functional, structural, diffusion, perfusion.*
Field strength	*Specify in Tesla*
Sequence & imaging parameters	*Specify the pulse sequence type (gradient echo, spin echo, etc.), imaging type (EPI, spiral, etc.), field of view, matrix size, slice thickness, orientation and TE/TR/flip angle.*
Area of acquisition	*State whether a whole brain scan was used OR define the area of acquisition, describing how the region was determined.*

Diffusion MRI ☐ Used ☐ Not used

Preprocessing

Preprocessing software	*Provide detail on software version and revision number and on specific parameters (model/functions, brain extraction, segmentation, smoothing kernel size, etc.).*
Normalization	*If data were normalized/standardized, describe the approach(es): specify linear or non-linear and define image types used for transformation OR indicate that data were not normalized and explain rationale for lack of normalization.*
Normalization template	*Describe the template used for normalization/transformation, specifying subject space or group standardized space (e.g. original Talairach, MNI305, ICBM152) OR indicate that the data were not normalized.*
Noise and artifact removal	*Describe your procedure(s) for artifact and structured noise removal, specifying motion parameters, tissue signals and physiological signals (heart rate, respiration).*
Volume censoring	*Define your software and/or method and criteria for volume censoring, and state the extent of such censoring.*

Statistical modeling & inference

Model type and settings	*Specify type (mass univariate, multivariate, RSA, predictive, etc.) and describe essential details of the model at the first and second levels (e.g. fixed, random or mixed effects; drift or auto-correlation).*
Effect(s) tested	*Define precise effect in terms of the task or stimulus conditions instead of psychological concepts and indicate whether ANOVA or factorial designs were used.*

Specify type of analysis: ☐ Whole brain ☐ ROI-based ☐ Both

Statistic type for inference<br>(See Eklund et al. 2016)	*Specify voxel-wise or cluster-wise and report all relevant parameters for cluster-wise methods.*
Correction	*Describe the type of correction and how it is obtained for multiple comparisons (e.g. FWE, FDR, permutation or Monte Carlo).*

Models & analysis

n/a	Involved in the study
☐	☐ Functional and/or effective connectivity
☐	☐ Graph analysis
☐	☐ Multivariate modeling or predictive analysis

Functional and/or effective connectivity	*Report the measures of dependence used and the model details (e.g. Pearson correlation, partial correlation, mutual information).*
Graph analysis	*Report the dependent variable and connectivity measure, specifying weighted graph or binarized graph, subject- or group-level, and the global and/or node summaries used (e.g. clustering coefficient, efficiency, etc.).*
Multivariate modeling and predictive analysis	*Specify independent variables, features extraction and dimension reduction, model, training and evaluation metrics.*
