# Supplementary figures and images for: Glutarate regulates T cell metabolism and anti-tumour immunity
Source: Nat Metab. 2023 Aug 21;5(10):1747–64. doi: 10.1038/s42255-023-00855-2 (PMC10590756; doi:10.1038/s42255-023-00855-2)

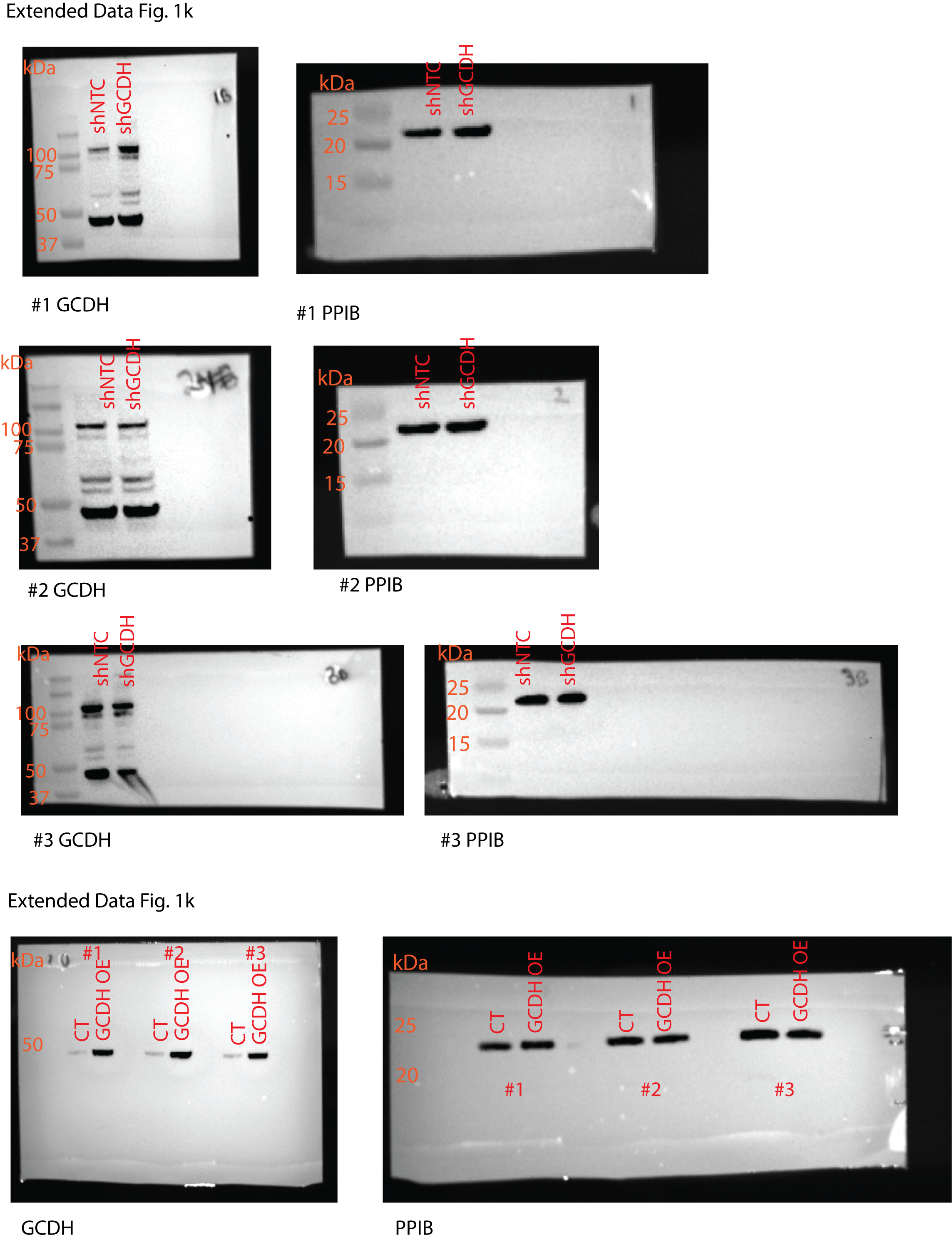

Supplement: Supplementary file 5 — Unprocessed western blots. [file 42255_2023_855_MOESM5_ESM.tif]

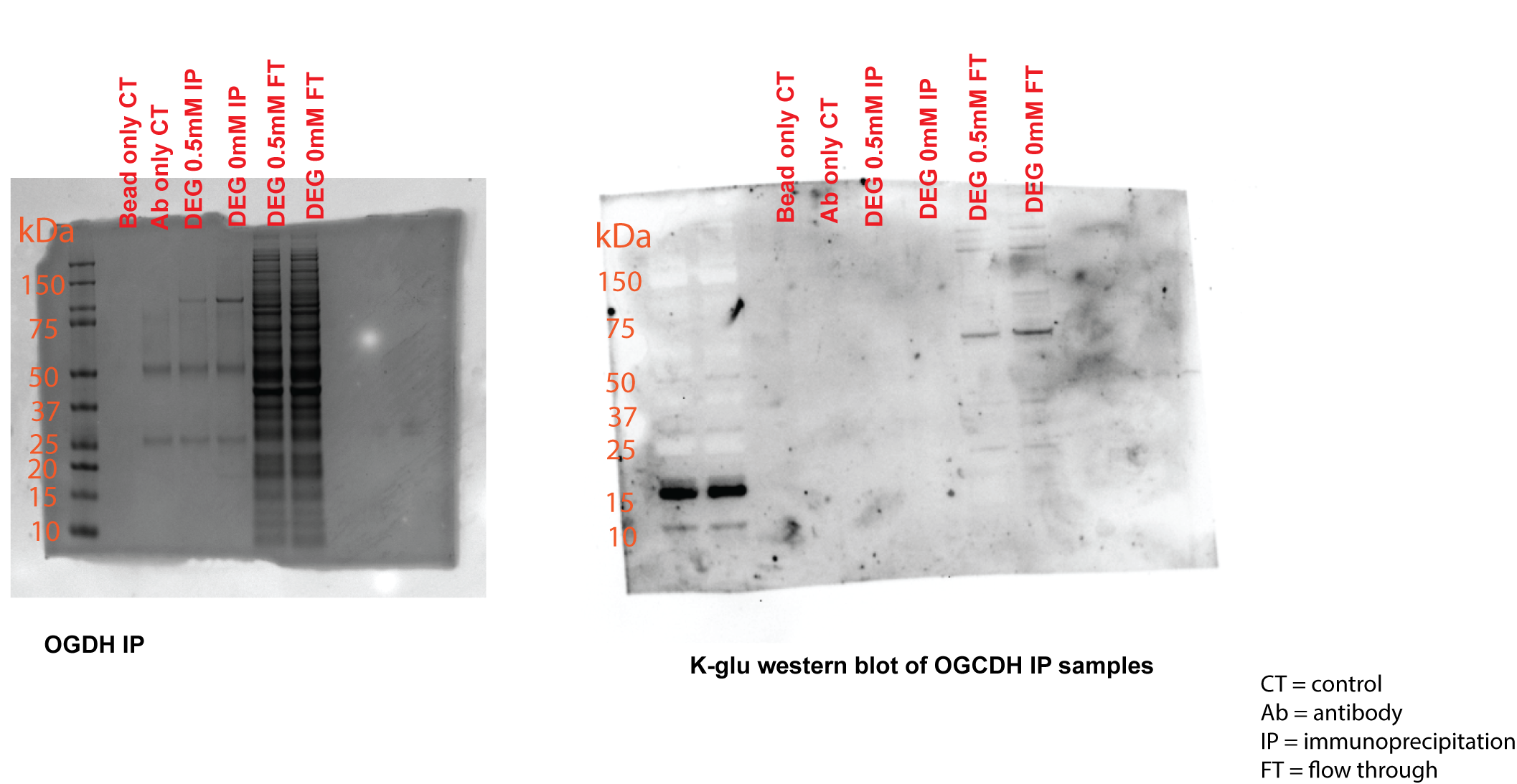

Supplement: Supplementary file 7 — Unprocessed western blots and gels. [file 42255_2023_855_MOESM7_ESM.tif]

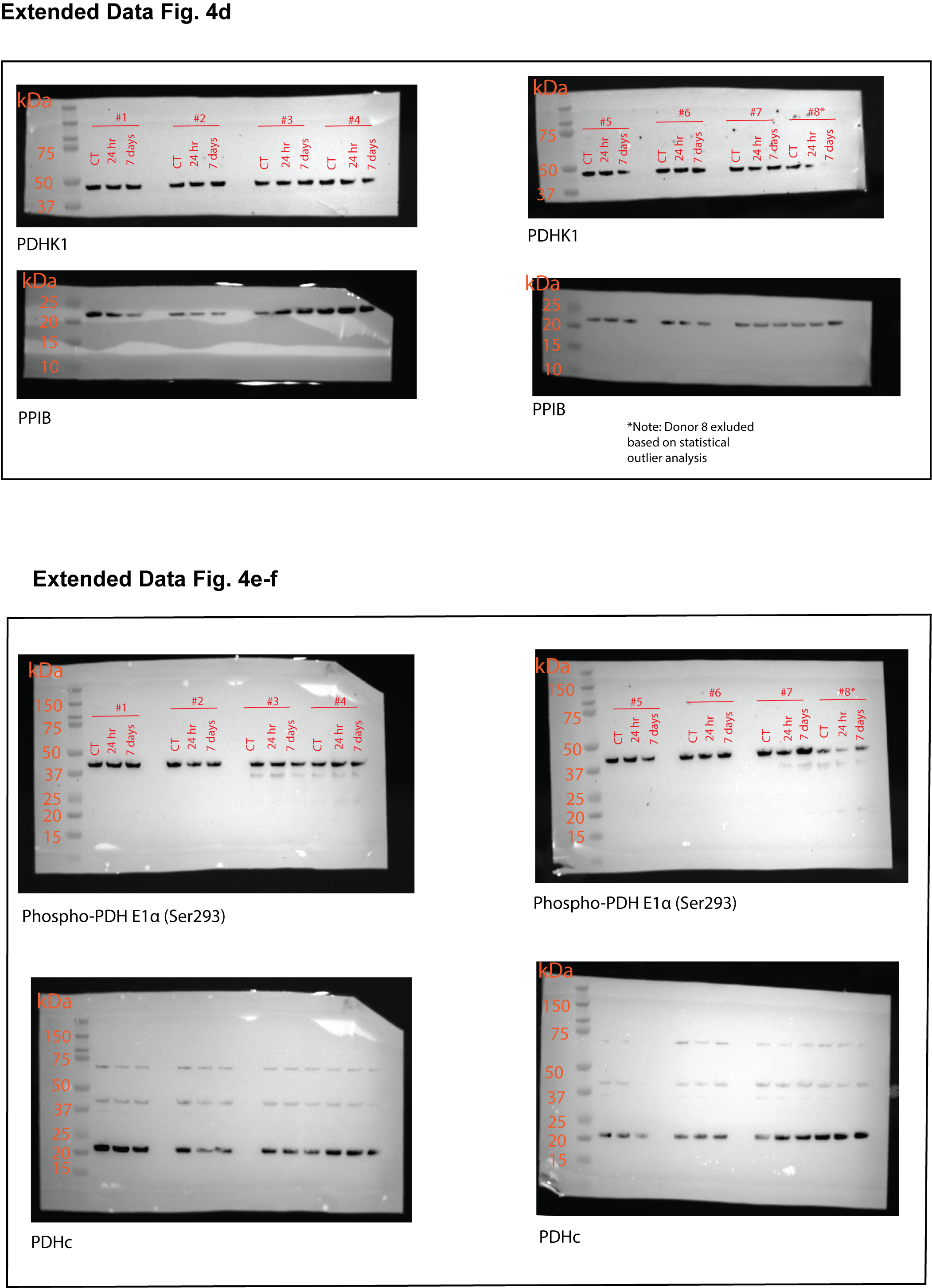

Supplement: Supplementary file 8 — Unprocessed western blots. [file 42255_2023_855_MOESM8_ESM.tif]
